# On Hunting Animals of the Biometric Menagerie for Online Signature

**DOI:** 10.1371/journal.pone.0151691

**Published:** 2016-04-07

**Authors:** Nesma Houmani, Sonia Garcia-Salicetti

**Affiliations:** 1 Télécom SudParis, Université Paris-Saclay, 9 rue Charles Fourier 91011 EVRY Cedex, France; 2 SAMOVAR, Télécom SudParis, CNRS, Université Paris-Saclay, 9 rue Charles Fourier 91011 EVRY Cedex, France; Nankai University, CHINA

## Abstract

Individuals behave differently regarding to biometric authentication systems. This fact was formalized in the literature by the concept of Biometric Menagerie, defining and labeling user groups with animal names in order to reflect their characteristics with respect to biometric systems. This concept was illustrated for face, fingerprint, iris, and speech modalities. The present study extends the Biometric Menagerie to online signatures, by proposing a novel methodology that ties specific quality measures for signatures to categories of the Biometric Menagerie. Such measures are combined for retrieving *automatically* writer categories of the extended version of the Biometric Menagerie. Performance analysis with different types of classifiers shows the pertinence of our approach on the well-known MCYT-100 database.

## 1. Introduction

Biometric systems’ performance is usually assessed *globally* on the whole available data, and that in terms of the two types of errors that a biometric system can make on a given sample: False Rejections and False Acceptances [1]. A False Rejection occurs when a genuine or authentic sample of a user is falsely rejected by the biometric system. A False Acceptance occurs when a forgery or imposture is falsely accepted by the system [1]. Even though system performance is measured *globally*, the difficulty in authenticating persons is not the same from one individual to another. In order to have a better insight on the behavior of a biometric system, an alternative is to identify groups of users having common traits and to assess performance on each group separately.

In [2,3,4,5,6], we introduced a personalized quality measure for online signatures, called Personal Entropy, which allows generating writer categories. Such categories showed a stable relative behavior when being confronted to several classifiers on different databases. This interesting result was later confirmed with numerous classifiers on large databases [7,8] in the framework of two international online signature verification competitions, namely BSEC’2009 [9] and ESRA’2011 [10].

For several other biometric modalities (speech, face, iris, fingerprints, keystroke dynamics), user groups have been formally defined and labeled with animal names that reflect their behavior regarding to biometric systems: this gave rise to the concept of “Biometric Menagerie” [11,12,13].

**Fig 1:**
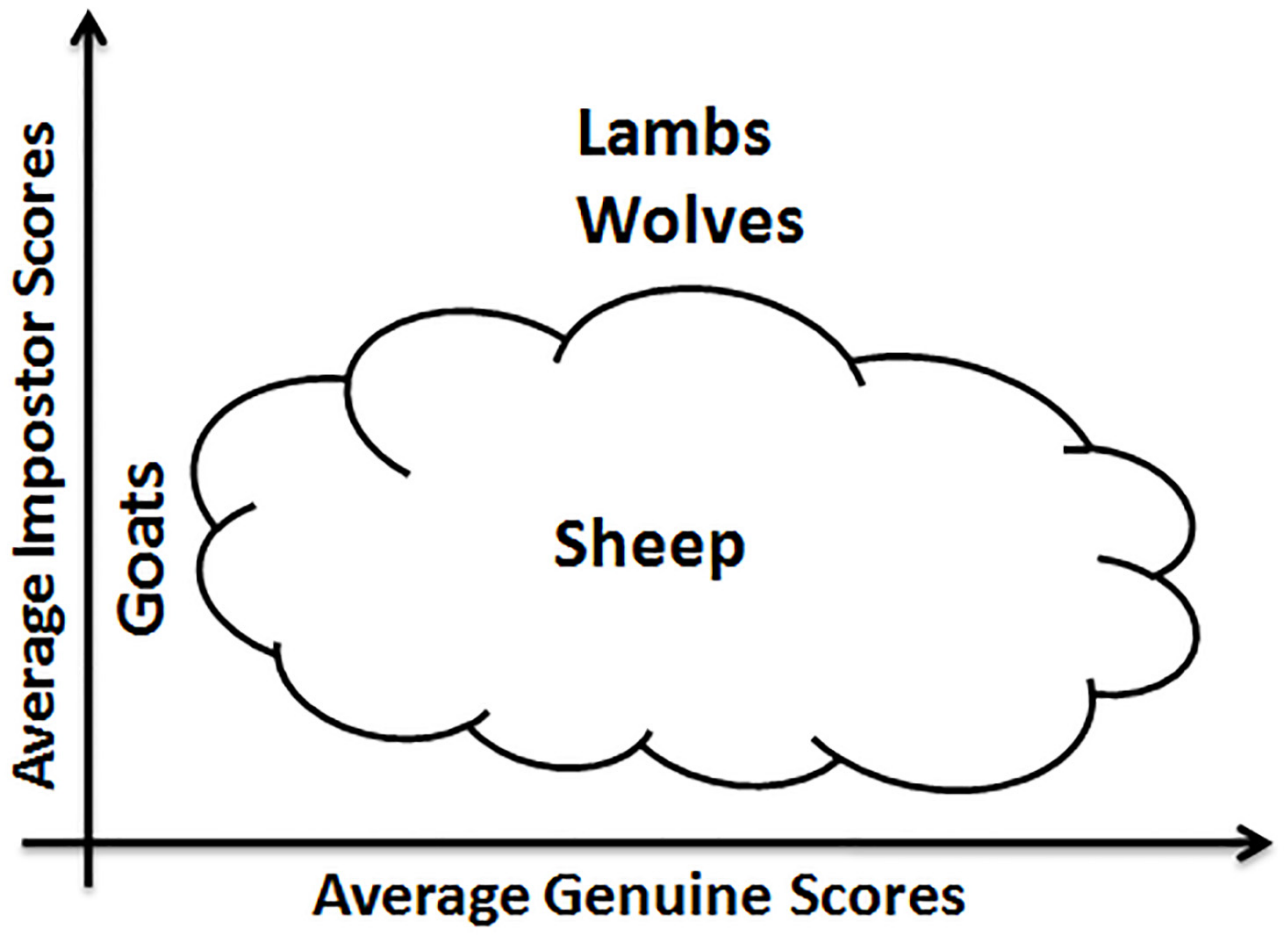
Doddington’s representation of the Biometric Menagerie [11].

The concept of “Biometric Menagerie” was initially formalized by Doddington et al. [11]. The authors grouped speakers in four non-exclusive categories, labeled as “*Sheep*”, “*Goats*”, “*Lambs*” and “*Wolves*” (Fig 1). Such categories are defined based on the genuine match scores *or* impostor match scores of a given classifier. “*Sheep*” represent speakers who are easy to recognize; they exhibit low FRR. At the opposite, “*Goats*” represent speakers who are difficult to recognize; they tend to increase the FRR. “*Lambs*” are speakers who are easy to imitate, leading to increase the FAR. Finally, “*Wolves*” are speakers who are successful at imitating others, tending to increase the FAR significantly. Such categorization was later applied in the context of face [12,14,15], fingerprint [12,13,15], and iris biometrics [12,15,16].

**Fig 2:**
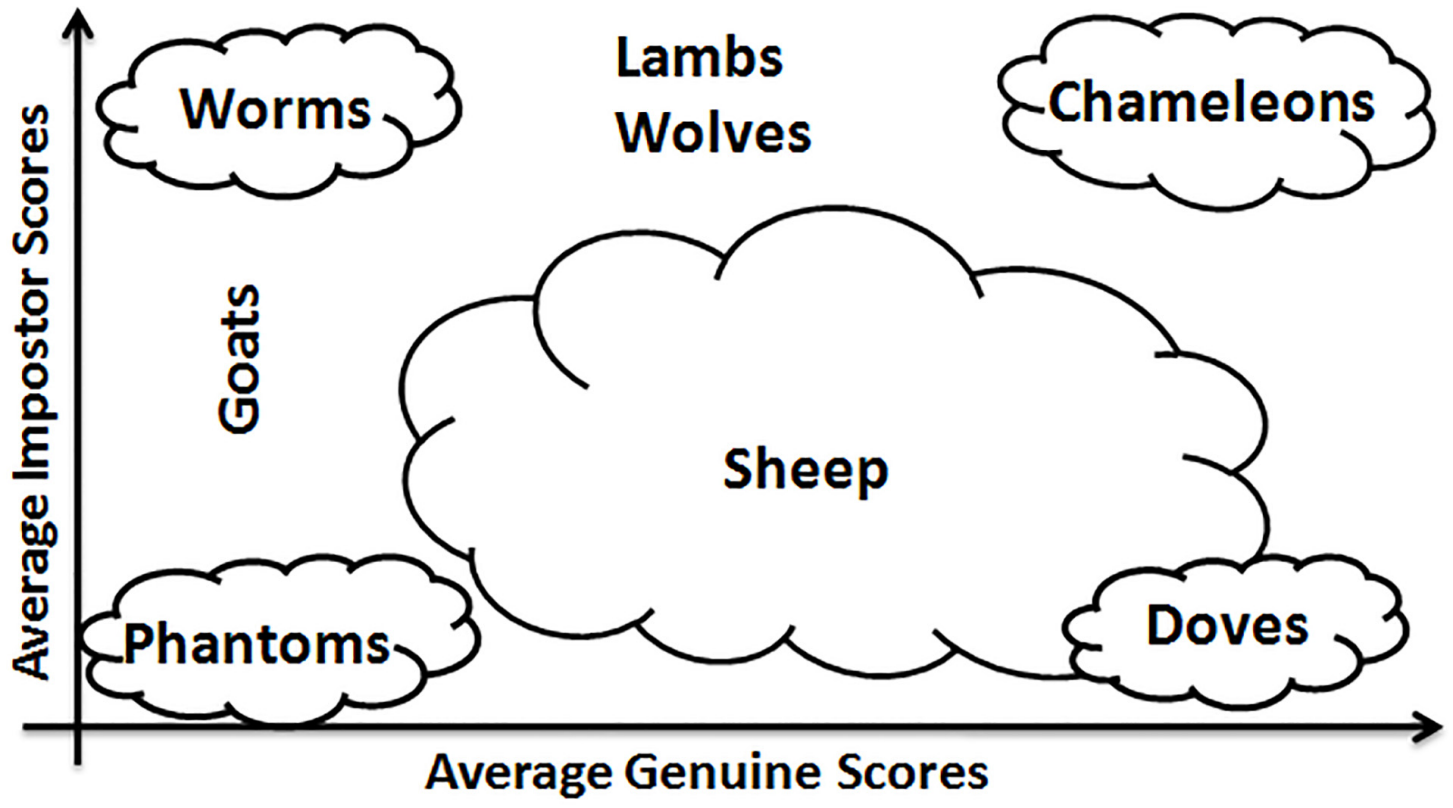
Yager & Dunstone’s representation of the Biometric Menagerie [12,13].

More recently, Yager & Dunstone [12,13] have completed such a Biometric Menagerie by adding four other categories of users: “*Worms”*, *“Chameleons”*, *“Phantoms”* and *“Doves”* (Fig 2). Such categories were defined according to a user’s *relationship* between the genuine and impostor match scores [12,13]. “*Chameleons*” lead to high genuine and impostor match scores. At the opposite, “*Phantoms*” lead to low genuine and impostor match scores. “*Doves*” are a sub-group of “*Sheep”*; they represent the best users since they lead both to high genuine and low impostor match scores. At the opposite, “*Worms*” are a sub-group of “*Goats”;* they are the worst users, showing low genuine and high impostor scores. This categorization was applied on face, speech, fingerprint, iris and keystroke modalities [12].

Different works focusing on Doddington’s categorization [11,12,13,14,15,16,17,18,19] provide evidence that the Biometric Menagerie exists in most biometric modalities. To our knowledge, in the signature biometrics, there is no complete study on the existence of all the categories of the Biometric Menagerie. A preliminary study in offline signature pointed out the existence of “*Goats*” [20] and their variations according to different feature extractions. Another study on revocability in online signature analyzed the stability of individuals in the categories of “*Goats*” and “*Sheep*” [21].

The lack of works in the handwritten signature literature on the Biometric Menagerie is quite surprising since additionally to our works [2,3,4,5,6], the concept of writer categories was already addressed in several other works on handwritten signature [22,23,24,25,26,27]. By relating writer categories to classifier performance, such works pointed out that writers exhibit different behaviors regarding to signature verification systems. Indeed, they set some trends on classifier performance according to *quality criteria* of signatures, namely complexity, variability and legibility. In the case of offline signature, Alonso et al. in [22,23] generated manually three groups based on *writer’s intra-variability*, which was measured using Mahalanobis distance [22,23]. When analyzing such categories, the authors observed that performance was degraded for writers with highly variable signatures. In [23,24], Alonso et al. considered *complexity* and *legibility* criteria for categorizing writers visually into four groups. The authors noted that the most complex and legible signatures lead to the best classifier performance. In the case of online signature, Brault et al. [25] concluded that “*problematic signers”* are those having *unstable* and *not complex* enough signatures [25].

All these works agree on specific quality criteria for signatures (complexity, stability and legibility) that predict a certain performance level of signature verification systems. However, none of these works tackles the Biometric Menagerie for signatures.

In this paper, we aim at relating the above-mentioned quality criteria for signatures to the concept of Biometric Menagerie. In our previous works [2,4,5,6], we introduced a quality measure, called Personal Entropy, based on the concept of entropy, widely exploited in data mining [28,29,30,31]. The proposed Personal Entropy measure quantifies directly on genuine signatures both their *complexity* and their *stability*. This work will show that Personal Entropy can be successfully exploited for studying the Biometric Menagerie.

Additionally, the objective of this work requires assessing the vulnerability of a writer to skilled forgeries. Indeed, user groups of the Biometric Menagerie are traditionally defined in terms of average genuine and/or average impostor classifier output scores. For this reason, we propose another personalized quality measure, namely *Relative Entropy*, which allows a writer to be characterized not only in terms of signature complexity and signature variability, as Personal Entropy does, but also in terms of *how difficult it is to attack* such a signature with a *skilled forgery*. In the literature, to our knowledge, the only study tackling this problem is that of Brault et al. [26]. In such work, the difficulty of reproducing a signature, namely of carrying out a skilled forgery, is quantified by a “*difficulty coefficient*” related to *complexity* of the signature [25]. It is computed as a function of the rate of geometric modifications per unit of time. The study concludes that *“problematic signers”* in terms of systems’ performance are those having a low “difficulty coefficient”.

The adopted methodology in this paper is to hunt animals of the Biometric Menagerie in online signature by combining the two entropy-based quality measures. Moreover, our proposal is to retrieve groups of users *automatically* with a *clustering procedure* performed on such two measures, and to analyze the relationship existing between the characteristics of the obtained groups and classifier performance. The originality of our contribution is to offer for online signature an alternative to the usual methodology for hunting categories of the Menagerie, methodology that has the limitation of being dependent on a given classifier’s output scores, as stated by several works in the literature [12,13,15,16,17,18,19,20].

This paper is organized as follows: in Section 2, we propose to retrieve automatically by Hierarchical Clustering writer categories of the Biometric Menagerie, based on the output scores of local and global classifiers. In Section 3, we describe the two entropy measures and study their relationship to verification performance. In Section 4, writer categories of the Biometric Menagerie are retrieved by Hierarchical Clustering, now based on the two entropy measures. Conclusions and perspectives are given in Section 5.

## 2. Looking for the Menagerie by Output Scores’ Analysis

In this section, we intend to retrieve writer categories of the Biometric Menagerie on MCYT-100 online signature database [32], and that by exploiting two classifiers: the first is based on Dynamic Time Warping (DTW) that matches two signatures *locally* [33]; the second relies on Hamming distance that performs a *global* match of two signatures. The aim of this experiment is to illustrate for online signature the statement of Yager & Dunstone [12]: “*a person cannot be labeled an animal independent of a specific algorithm*”.

### 2.1. MCYT-100 database description

In this work, we used the widely used and freely available MCYT-100 database [32]. In such database, signatures are acquired on WACOM pen tablet, model INTUOS A6 USB. The pen tablet resolution is 2540 lines per inch, and the precision is 0.25mm. The maximum detection height is 10 mm (pen-up movements are also considered), and the capture area is 127 mm (width) × 97 mm (height). The sampling frequency was set to 100 Hz. The capture area was further divided into 37.5 mm (width) × 17.5 mm (height) blocks which are used as frames for acquisition. At each sampled point of the signature, the digitizer captures pen coordinates, pen pressure (1024 pressure levels), and Azimuth and Altitude pen inclination angles.

Signature corpus contains genuine and shape-based highly skilled forgeries with natural dynamics. In order to obtain the forgeries, each donor is requested to imitate other signers by writing naturally, without artifacts such as breaks or slowdowns. The acquisition procedure is as follows. Signer *S*_*i*_ writes a set of 5 genuine signatures, and then 5 skilled forgeries of the signer *S*_*i-1*_. This procedure is repeated four more times imitating previous users *S*_*i-2*_, *S*_*i-3*_, *S*_*i-4*_ and *S*_*i-5*_. As a result, each signer contributes with 25 genuine signatures in 5 groups of 5 signatures each, and is forged 25 times by 5 different forgers. The total number of donors in MCYT is 330 [32]. However, a subset of only 100 persons is freely available (MCYT-100).

### 2.2. Score computation of the two classifiers

In this work, we used the DTW-based classifier considering the raw coordinates as input data. It has been shown in different online signature competitions that pen coordinates are sufficient for obtaining good performance with DTW classifier [34]. Concerning the other classifier, we used 40 features [35] computed in a holistic manner on each signature sample. Indeed, it is well-known in the literature [36] that a global approach for signature matching requires a large amount of features for building a good enough classifier.

Genuine and impostor scores are computed as follows with each classifier: we carry out 5 random samplings on genuine signatures. Each sampling contains 5 genuine signatures considered as enrolment signatures. Among the remaining genuine signatures, 10 are used for computing genuine scores. Among the available skilled forgeries, 15 are used for computing impostor scores. Finally, the FRR and the FAR are averaged on the 5 random samplings.

The dissimilarity matching score of the DTW-based classifier is defined as:

$$Score=D_{\text{min}}(probe,enrolment_{i})i=1,\ldots ,5$$ (1)

where *D*_min_ denotes the minimum of the five distances computed between the probe signature and the five enrolment signatures.

The dissimilarity matching score of the global classifier is defined as:

$$Score=HD_{avg}(probe,enrolment_{i})i=1,\ldots ,5$$ (2)

where *HD*_*avg*_ denotes the average of the five Hamming distances computed between the probe signature and the five enrolment signatures.

The degree of dissimilarity between two signatures is measured by this dissimilarity score. A genuine or authentic score results of matching two signatures of the same user; a forgery or impostor score results of matching two signatures belonging to two different users. The decision rule of the biometric system is the following: when the dissimilarity score is lower than a given threshold value, the probe sample is accepted (the claimed identity too); otherwise it is rejected. A genuine score higher than the decision threshold results in a False Rejection. An impostor score lower than the threshold results in a False Acceptance [1]. The False Acceptance Rate (FAR) is defined as the fraction of impostor scores that are lower than the threshold; the False Rejection Rate (FRR) is defined as the fraction of genuine scores that are higher than the threshold. For each value of the decision threshold a couple of error rates (FRR, FAR) is computed. A biometric system is usually evaluated in the literature by plotting the FRR against the FAR, when computed at different values of the threshold; this results in the Detection Error Tradeoff (DET-Curve) that displays the tradeoff between the FRR and the FAR [1].

### 2.3. Hunting animals of the Menagerie with local and global classifiers

Animals of Doddington’s Menagerie (Fig 1) depend on either the FRR or the FAR: “*Goats”* and “*Sheep”* are characterized by *genuine scores* (writers respectively difficult or easy to recognize); “*Lambs”* and “*Wolves”* are defined in terms of *impostor* scores (writers respectively easy to imitate or successful at imitating). Besides, in Yager & Dunstone’s Menagerie (Fig 2), new animal groups were defined in terms of a *relationship between genuine and impostor* scores.

**Fig 3:**
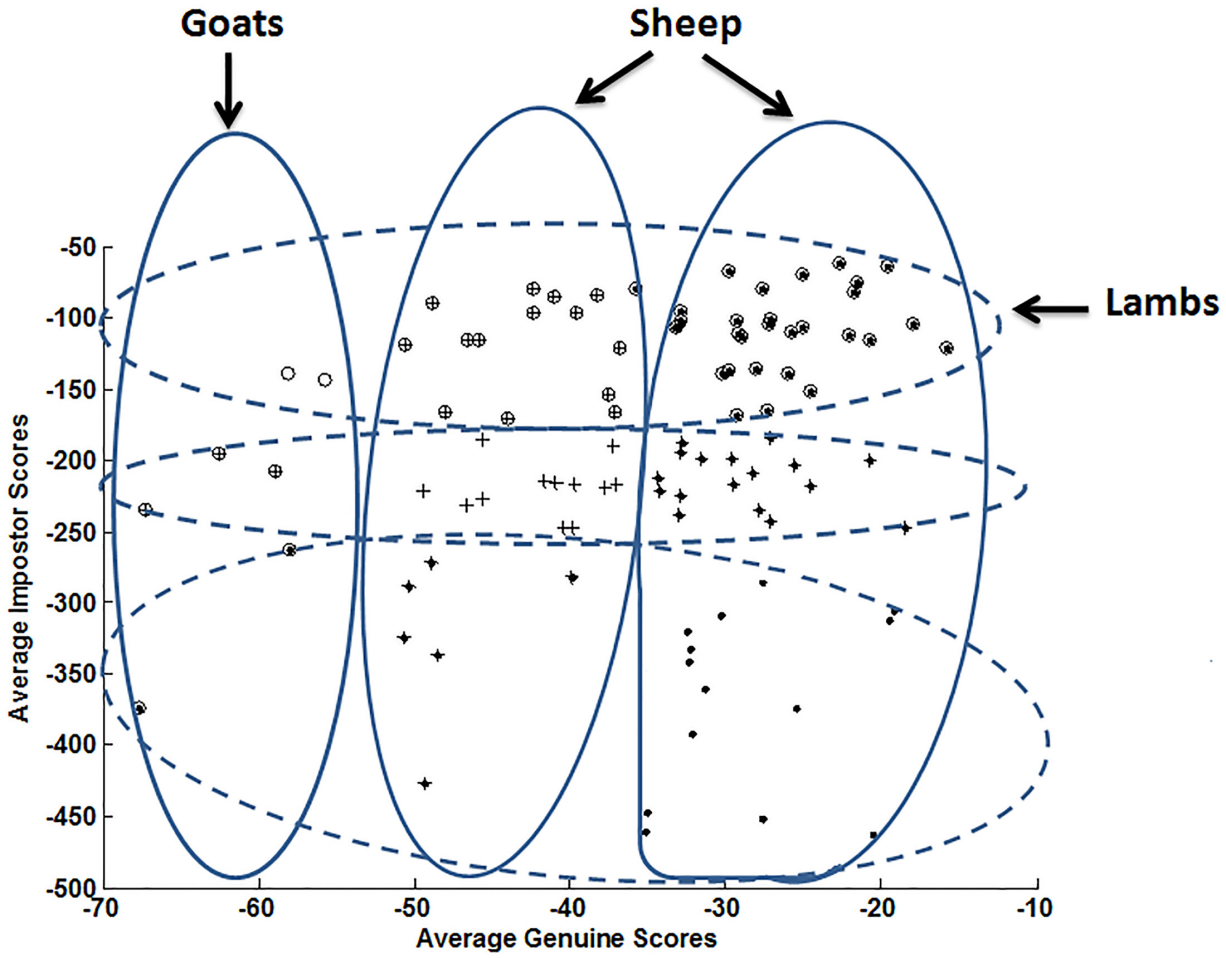
Animals of the Biometric Menagerie hunted with the local classifier.

**Fig 4:**
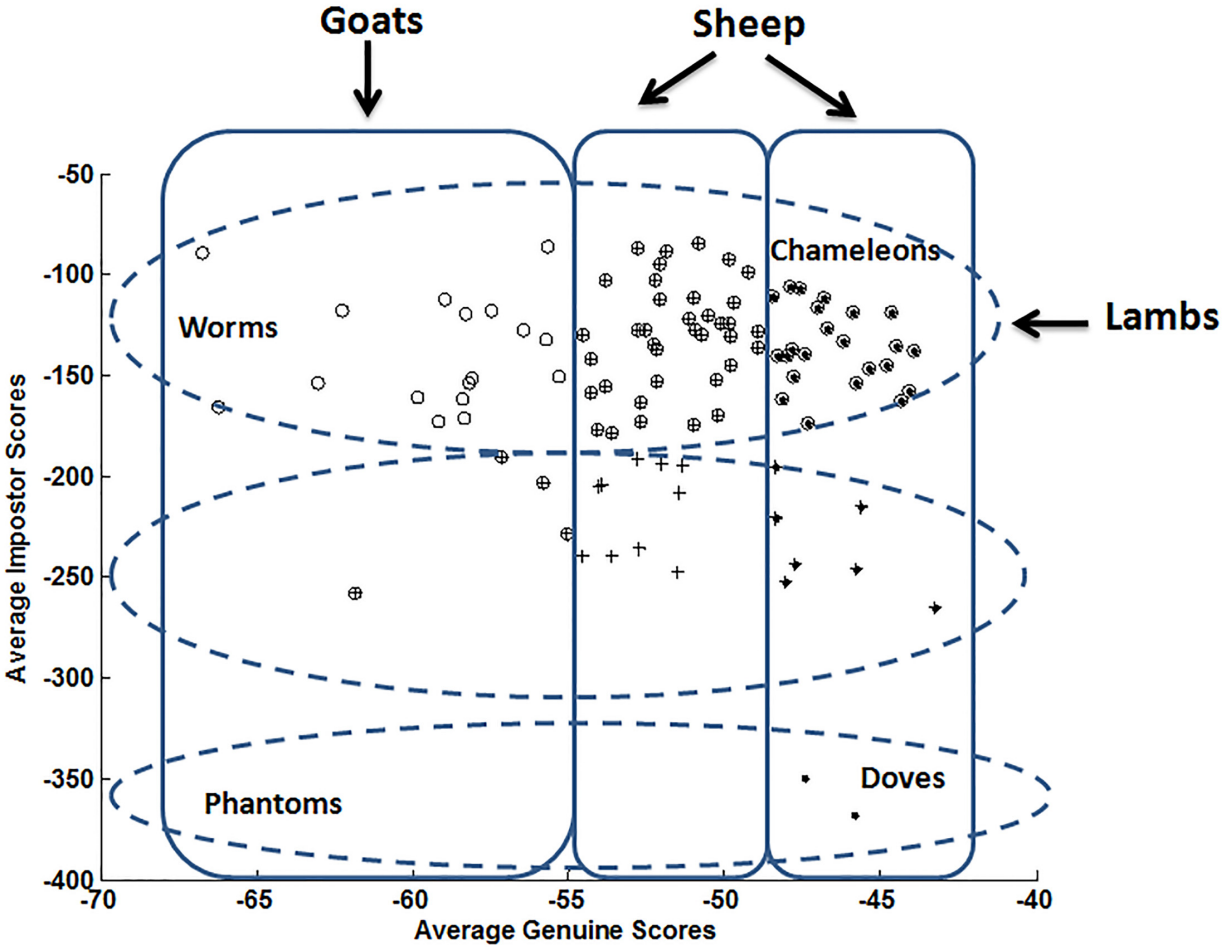
Animals of the Biometric Menagerie hunted with the global classifier.

Figs 3 and 4 display writer categories of the Biometric Menagerie when considering respectively the local and the global classifiers. Such categories are obtained automatically by performing a Hierarchical clustering [37,38] first on genuine output scores, then on impostor output scores. Thus, three categories are retrieved along the genuine scores’ axis and 3 categories along the impostor scores’ axis. It is worth noticing that no thresholds are involved in the clustering procedure leading to animal groups. The resulting membership of users to animal groups is represented in Figs 3 and 4 by contours of arbitrary shape.

We notice as expected [12,13,15,16] that the resulting categories of the Menagerie strongly depend on the classifier. Indeed, with the global classifier, there are significantly more “*Worms*” (writers difficult to recognize) and more “*Lambs*” (writers easy to imitate) than with the local classifier. On the other hand, with the local classifier, there are significantly more “*Doves*” (writers easy to recognize). All such remarks are in perfect accordance with the literature [36]: it has been shown that classifiers based on a local paradigm outperform in general those based on a global one. The above analysis naturally leads to the following conclusion: *the retrieved writer categories reflect the characteristics of the classifier that is used*. Although this result is not surprising, since the Biometric Menagerie is precisely defined relying on classifiers’ scores [12], it points out an inherent methodological difficulty for the retrieval of writer categories.

The present work responds to this methodological difficulty by proposing an alternative approach relying on quality measures. Two entropy measures are exploited for this task: first, “Personal Entropy”, efficient in former works [2,3,4,5,6] for retrieving meaningful writer categories in terms of verification performance, by using only *genuine* data; second, a “Relative Entropy” measure here introduced for measuring signature vulnerability to skilled forgeries, computed with *both genuine and impostor data*. Next section shows that those measures allow characterizing signatures with regard to their behavior in terms of FRR and FAR, with both the local and global classifiers above presented.

## 3. Relating Quality Measures to Verification Performance

For characterizing a writer, we propose on one hand our former Personal Entropy measure [4,6] computed on genuine signatures and, on the other hand a Relative Entropy measure computed on both genuine signatures and *skilled forgeries*. Note that a specific trait of the signature biometrics, as it is behavioral, is that forgeries in the literature are since a long time *“skilled”*, namely that the impostor tries to match as much as possible the shape or even the dynamics of the target signature.

Our aim in this section is to show that Personal Entropy characterizes a writer in terms of the FRR, while Relative Entropy does the same in terms of a *tradeoff* between FRR and FAR.

In the following, we first detail how Personal Entropy and Relative Entropy are computed. In Section 3.2, writer categories are retrieved automatically with each measure. Finally, in Section 3.3, the behavior of such categories is studied in terms of classifier performance.

### 3.1. Recalling a Writer’s Personal Entropy

As detailed in [4,6], for a given writer, Personal Entropy is measured by means of local density estimation after training a Hidden Markov Model (HMM) [33] on a set of *K* genuine signatures. As in our former works, signatures are described only by their raw coordinates *(x*,*y)*.

A random variable *Z*_*i*_ is associated to each stationary portion *S*_*i*_ of the signature, generated by the Viterbi algorithm [33] according to the Writer-HMM. The number of portions *N* is the number of states of the writer-HMM, computed according to the length of genuine signatures as follows:

$$N=\frac{T_{Total}}{4*30}$$ (3)

where, *T*_*Total*_ is the total number of sampled points available in the genuine signatures, and *M = 4* is the number of Gaussian components per state.

Then, the entropy [28,29] of a portion *S*_*i*_ is defined as that of an ensemble of outcomes of *Z*_*i*_, as follows:

$$H(Z_{i})=-\sum_{z\in S_{i}}p(z){\text{log}}_{2}\;p(z)$$ (4)

where z corresponds to a given point in the signature described by its coordinates *(x*,*y)*, belonging to the current portion *S*_*i*_ according to the Writer-HMM.

The local probability distribution function is estimated by using all the sample points belonging to each portion across the *K* instances of the writer’s signatures. Then, the entropy of each genuine signature is computed by averaging the local entropy values H(Z_i_) on all of its *N* portions *S*_*i*_, and then by normalizing with signing time *T* of the genuine signature:

$$H(Z)=\frac{1}{N*T}\sum_{i=1}^{N}H(Z_{i})$$ (5)

Finally, by averaging this measure *H(Z)* across the writer’s *K* training signatures, we obtain a writer’s Personal Entropy [4,6].

### 3.2. Measuring a Writer’s Relative Entropy

Relative Entropy measures the Kullback-Leibler distance between two probability distributions [28]. We propose in this work to measure, for a writer, the Kullback-Leibler distance between the local probability laws of his/her genuine signatures and that of his/her skilled forgeries. This measure aims at characterizing a writer in terms of the relative behavior existing between his/her genuine signatures and his/her skilled forgeries.

For computing Personal Entropy, the local density is estimated after training an HMM only on a set of genuine signatures. Now, for computing Relative Entropy, we use *two Writer-HMMs*: one for estimating local probability density functions (PDFs) of genuine data, and the second for estimating local PDFs of skilled forgeries. The number of states of the two HMMs is not the same since each one depends on the average length of their associated training signatures (Eq 3).

In other words, for each writer, we build a Writer-HMM with *K* genuine signatures and a Writer-HMM with *K* skilled forgeries. Then, we compute the symmetric version of Kullback-Leibler distance [28] for such a writer considering his/her *K* genuine signatures and *K* skilled forgeries as well. On each genuine signature *i* and on each skilled forgery *j*, we compute:

$$D_{gen_{i}^{}}(p,q)=\frac{1}{2}\left[\sum_{z\in gen_{i}}p(z){\text{log}}_{2}\frac{p(z)}{q(z)}+\sum_{z\in gen_{i}}q(z){\text{log}}_{2}\frac{q(z)}{p(z)}\right]$$ (6)

$$D_{sk_{j}}(p,q)=\frac{1}{2}\left[\sum_{z\in sk_{j}}p(z){\text{log}}_{2}\frac{p(z)}{q(z)}+\sum_{z\in sk_{j}}q(z){\text{log}}_{2}\frac{q(z)}{p(z)}\right]$$ (7)

where *p*(*z*) is the local probability density value on point *z* = (*x*,*y*) belonging to the current portion according to the Writer-HMM of genuine signatures; *q*(*z*)is the local probability density value on point *z* = (*x*,*y*) belonging to the current portion according to the Writer-HMM of skilled forgeries. Both summations are carried out on all points of respectively the *i*^*th*^ genuine signature *gen*_*i*_ and the *j*^*th*^ skilled forgery *sk*_*j*_.

Then, we average separately $D_{gen_{i}^{}}(p,q)$ across the *K* genuine signatures of the writer being considered and $D_{sk_{j}}(p,q)$ across his/her *K* skilled forgeries:

$$D_{gen}(p,q)=\frac{1}{K}\sum_{i=1}^{K}D_{gen_{i}}(p,q)$$ (8)

$$D_{sk}(p,q)=\frac{1}{K}\sum_{j=1}^{K}D_{sk_{j}}(p,q)$$ (9)

Finally, we average both values of Eqs 8 and 9 with a simple mean rule:

$$D(p,q)=\frac{1}{2}\left[D_{gen}(p,q)+D_{sk}(p,q)\right]$$ (10)

Relative Entropy therefore measures for a given writer the Kullback-Leibler distance between the local probability laws of his/her genuine signatures *p* and that of his/her skilled forgeries *q*: *for a given writer*, *the lower such a distance*, *the more such writer’s signatures are vulnerable to skilled forgeries*.

### 3.3. Writer categories and verification performance

For avoiding the difficulty of choosing thresholds that would separate users into animal groups, we propose in this work performing a Hierarchical Clustering [37,38] on MCYT-100 database, on one hand with Personal Entropy and, on the other hand, with Relative Entropy. Three writer categories are thus automatically retrieved in each case after a study on the optimal number *k* of categories (see S1 Appendix). Indeed, different validity indices [39,40,41], considered as tools for evaluating quantitatively the results of the clustering algorithm, show that *k* = 3 is the optimal number of categories.

**Fig 5:**
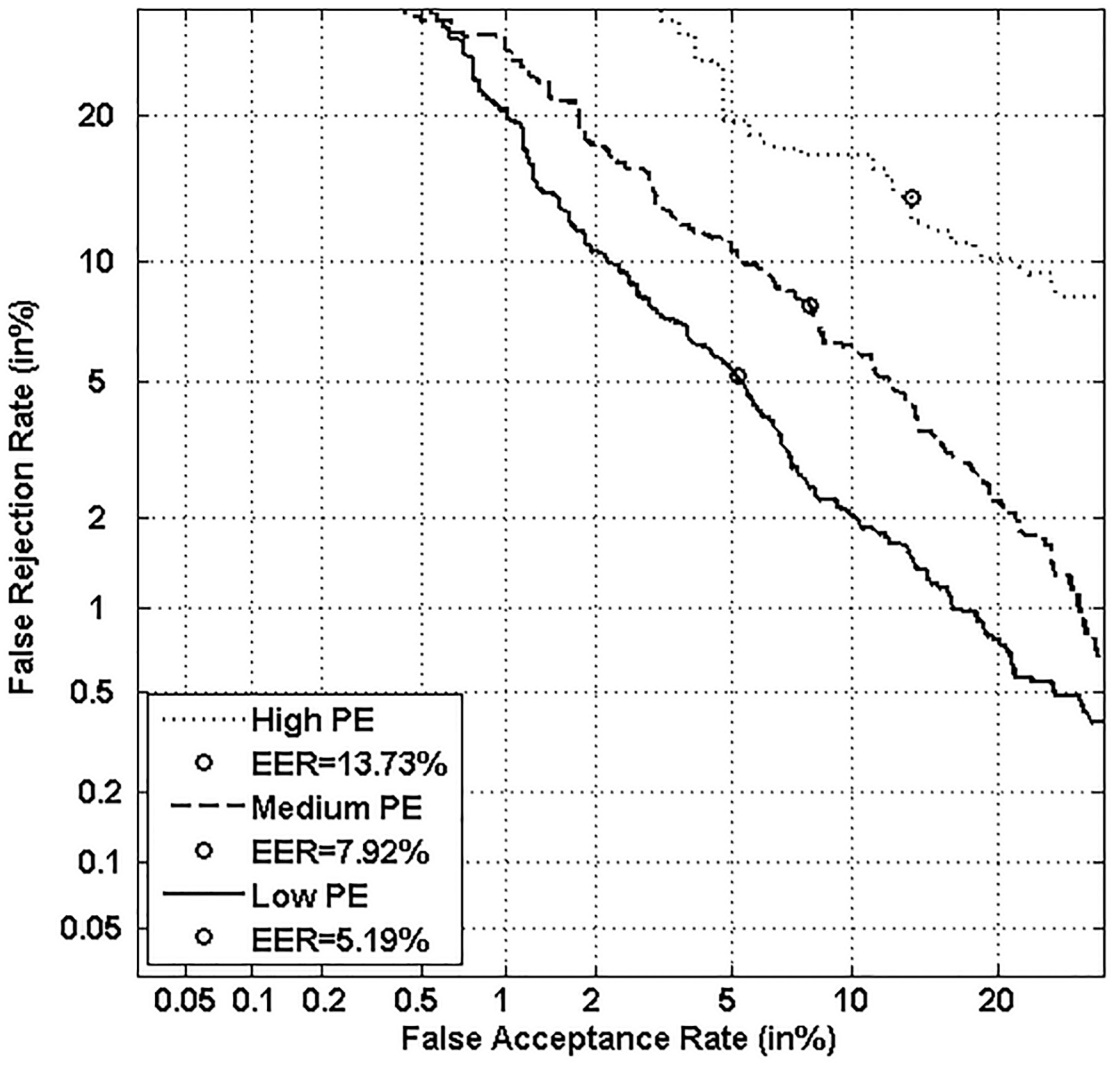
Performance of the local classifier on each category of MCYT-100 database generated with Personal Entropy (PE).

**Fig 6:**
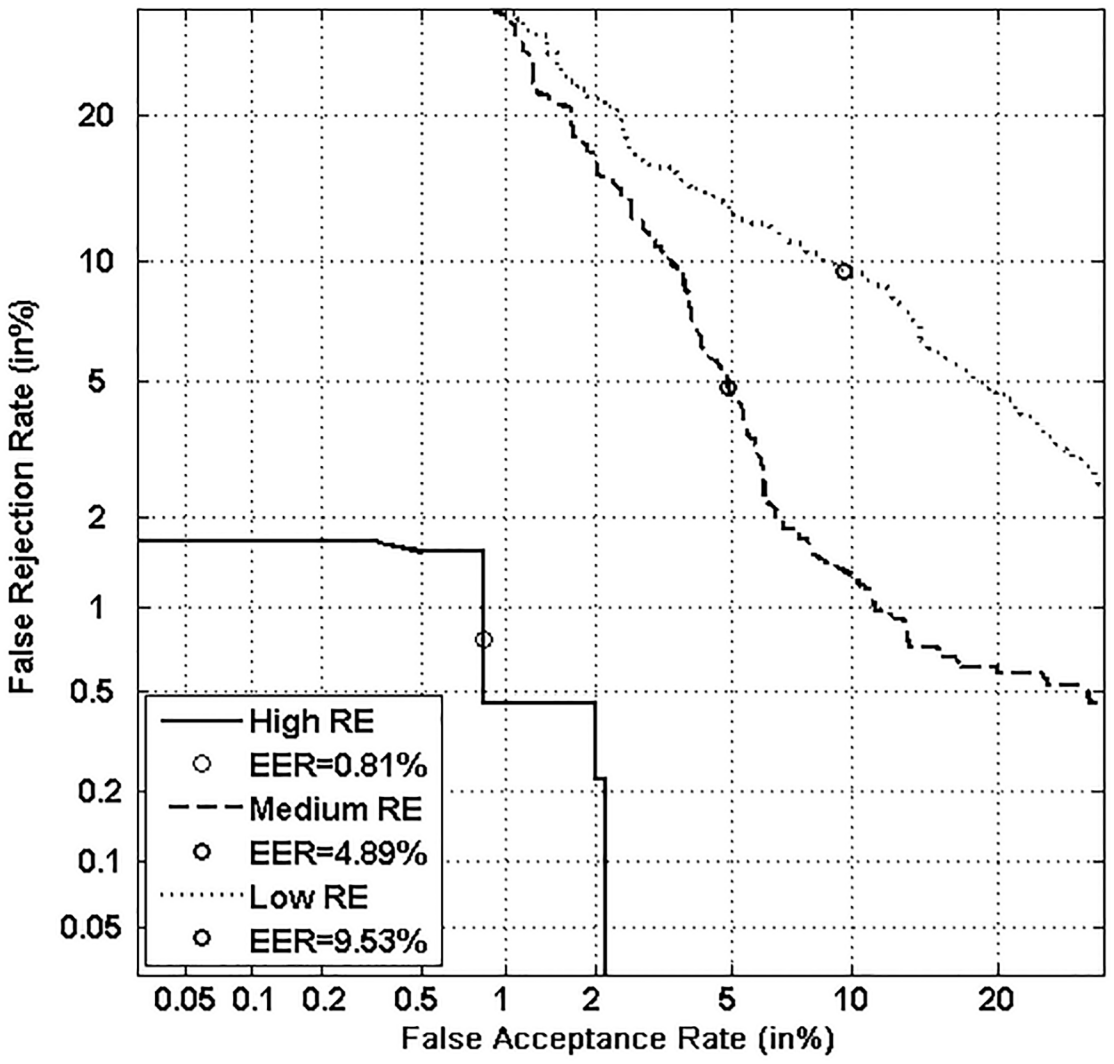
Performance of the local classifier on each category of MCYT-100 database generated with Relative Entropy (RE).

**Fig 7:**
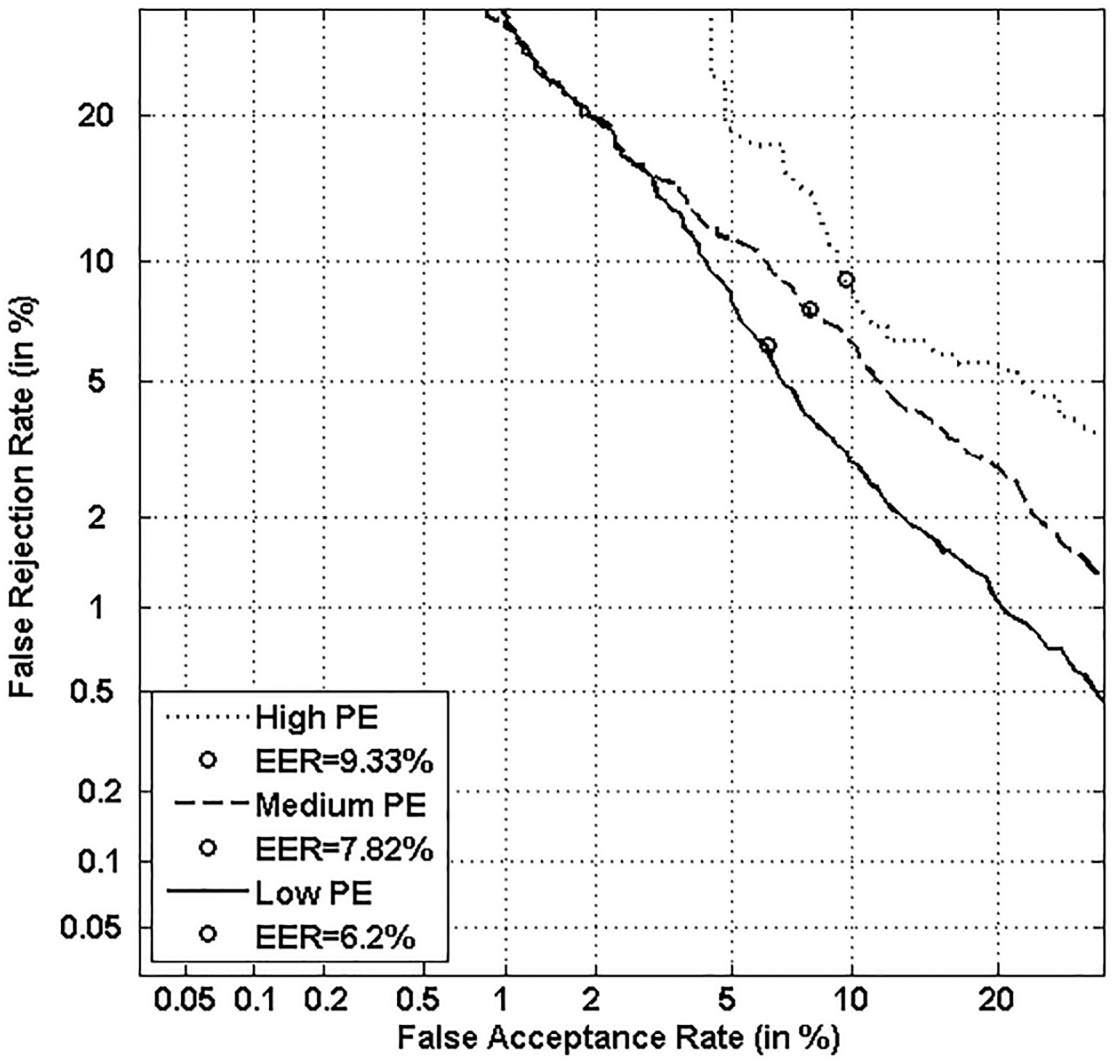
Performance of the global classifier on each category of MCYT-100 database generated with Personal Entropy (PE).

**Fig 8:**
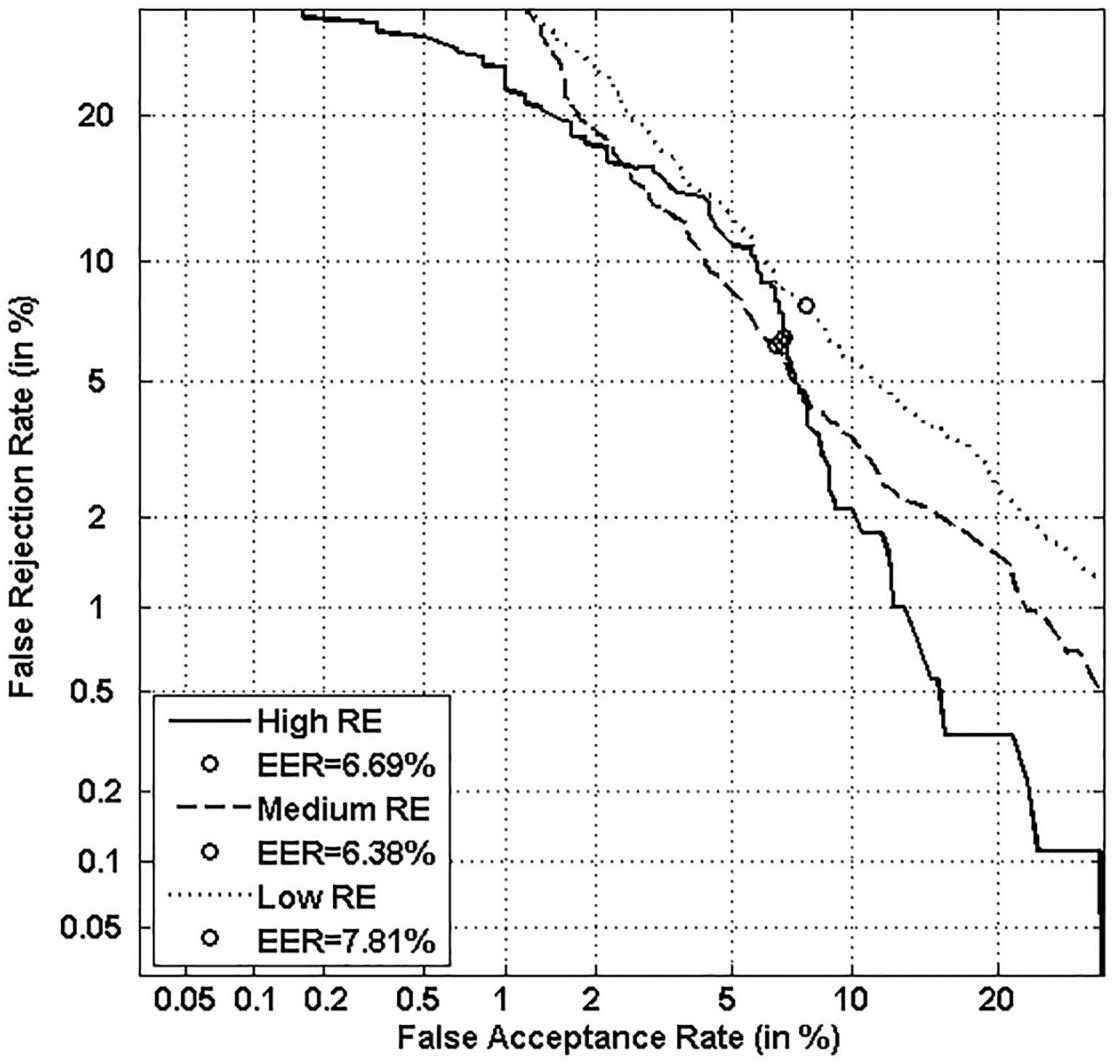
Performance of the global classifier on each category of MCYT-100 database generated with Relative Entropy (RE).

Figs 5, 6, 7 and 8 respectively show verification performance obtained with the local classifier (Figs 5 and 6) and the global classifier (Figs 7 and 8), and that on the three categories generated with Personal Entropy and on the other three categories generated with Relative Entropy.

We first remark that there is a difference in performance between the three writer categories, obtained with each entropy measure. Also, there is a significant difference in performance between the best category obtained with Personal Entropy and the best obtained with Relative Entropy. For the local classifier, Figs 5 and 6 show a relative improvement in performance of 91.50% at the EER on the best Relative Entropy category, which contains the least vulnerable signatures, comparatively to the best category generated with Personal Entropy. Improvement is observed not only at the EER but for all functioning points, especially for low values of FAR where FRR values are bounded at a quite low value (less than 2%). In case of the global classifier (Figs 7 and 8), the relative improvement between the two categories is only observed for high values of FAR and of FRR.

These results show that the two entropy measures generate categories containing writers exhibiting different properties. For this reason, we propose in the sequel to exploit such measures for analyzing writer categories of the Biometric Menagerie.

## 4. Hunting Animals of the Biometric Menagerie with Quality Measures

This section relates writer categories obtained with the two entropy measures to those of Doddington’s Menagerie (Fig 1) and Yager & Dunstone’s Menagerie (Fig 2).

Personal Entropy and Relative Entropy measures do not infer the same properties on writers, as reflected for example in Figs 5 and 6. Personal Entropy characterizes a writer through the local probability distribution of his/her genuine signatures. Therefore, it can be used to infer writer categories according to *FRRs*. On the other hand, Relative Entropy characterizes a writer in terms of his/her vulnerability to attacks: indeed, it is computed as the Kullback-Leibler distance between the local probability distribution of genuine signatures and that of skilled forgeries. For this reason, Relative Entropy can be used to infer writer categories in terms of *both FRRs and FARs*.

In this work, we exploit both entropy measures in a progressive manner: we start by analyzing Doddington’s Menagerie by means of Personal Entropy and Relative Entropy, considering them *separately*. Then, in a second step, we consider them *simultaneously* for analyzing Yager & Dunstone’s Menagerie.

### 4.1. Hunting animals of Doddington’s Menagerie

**Fig 9:**
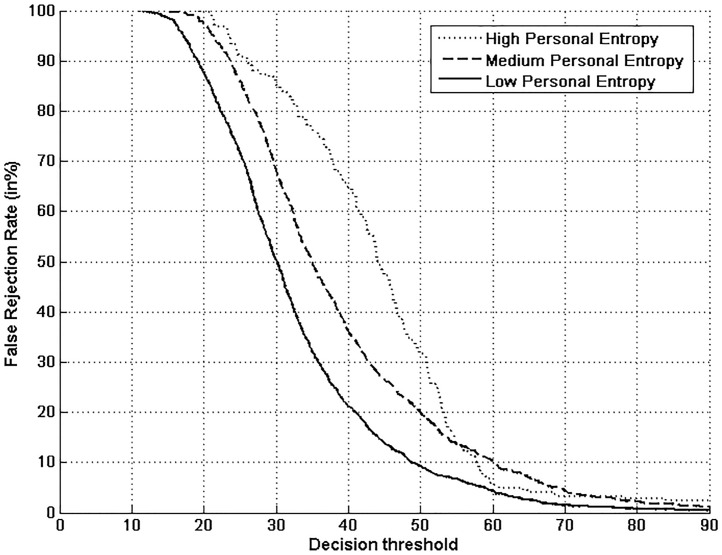
FRR with the local classifier on the three Personal Entropy categories.

**Fig 10:**
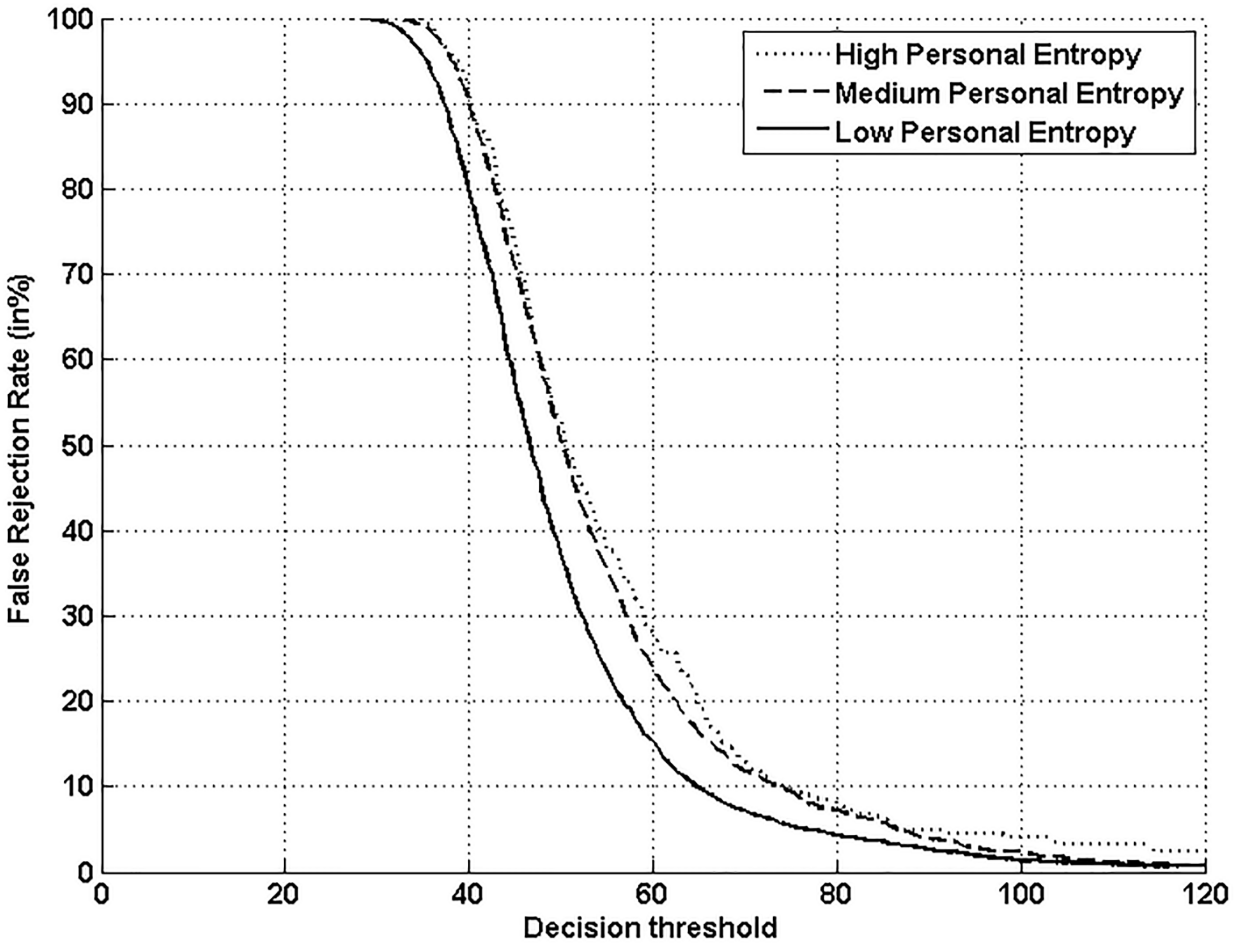
FRR with the global classifier on the three Personal Entropy categories.

**Fig 11:**
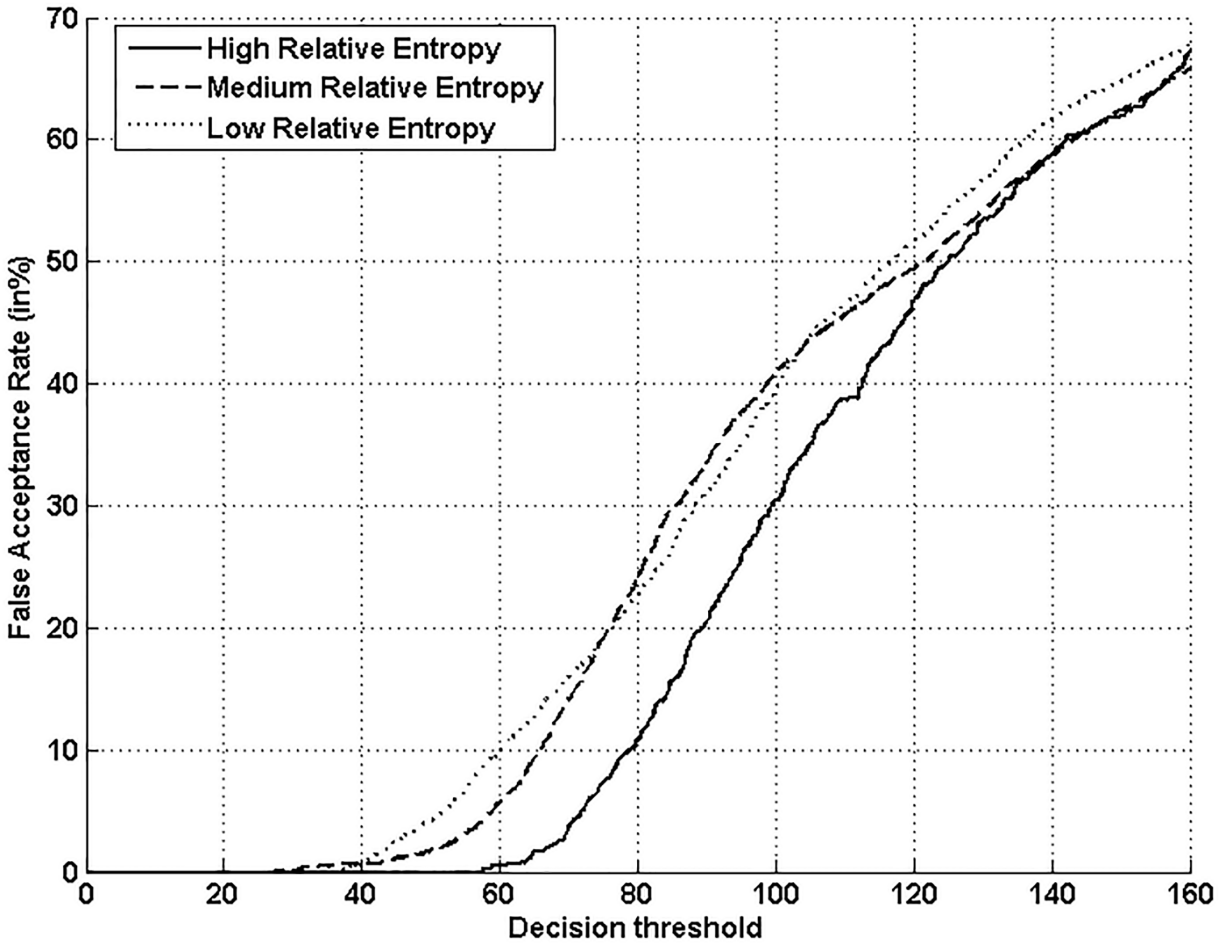
FAR with the local classifier on the three Relative Entropy categories.

**Fig 12:**
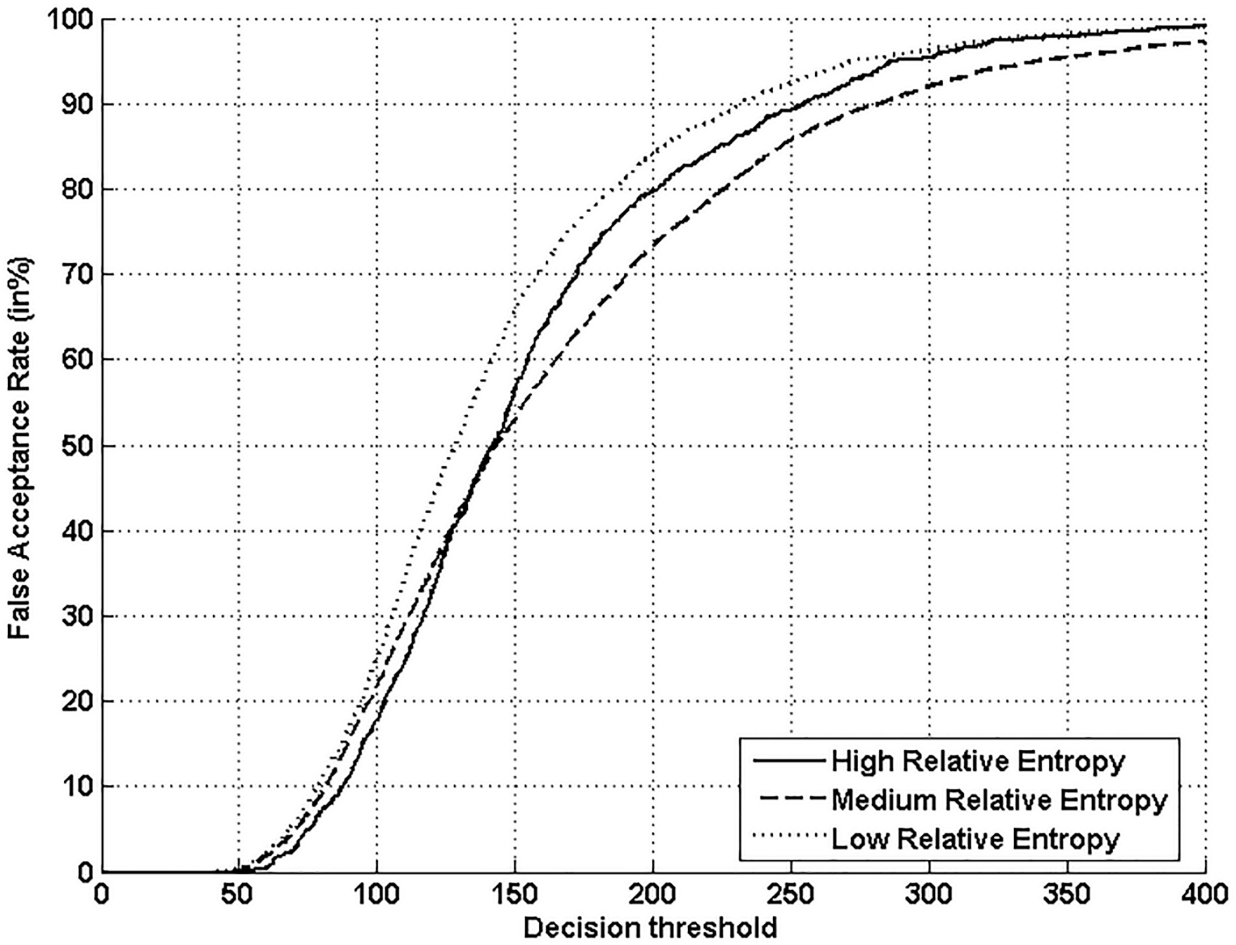
FAR with the global classifier on the three Relative Entropy categories.

In the following, we analyze on one hand the behavior of Personal Entropy-based categories in terms of FRR (Figs 9 and 10) and on the other hand the behavior of Relative Entropy-based categories in terms of FAR (Figs 11 and 12).

Figs 9 and 10 show that the highest FRR is obtained on the highest Personal Entropy category and the lowest FRR on the lowest Personal Entropy category. Finally, the medium Personal Entropy category exhibits FRR in between those of the two extreme categories. For these reasons, we are able to state that *Personal Entropy can be exploited for the automatic generation of meaningful writer categories in terms of FRR*.

**Fig 13:**
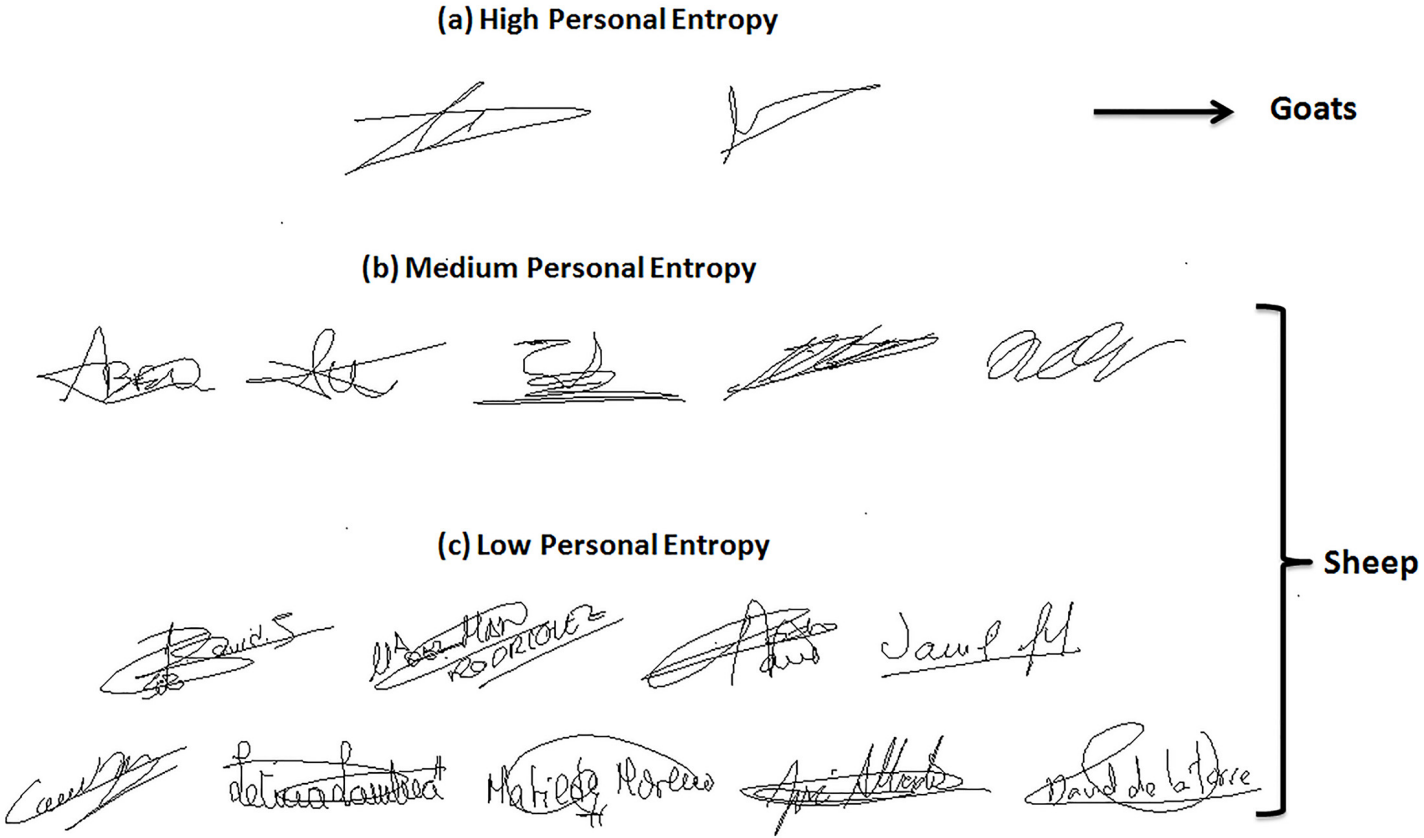
Signatures from MCYT-100 database of (a) High, (b) Medium and (c) Low Personal Entropy.

Based on this result, we propose to analyze the relationship between Personal Entropy-based categories and those categories of Doddington’s Menagerie that are only characterized by their average genuine scores: “*Goats*” and “*Sheep*”. “*Goats*” are actually defined as writers showing low genuine scores or accordingly high FRRs; in other words, such writers are difficult to recognize, and thus *correspond to writers of the highest Personal Entropy category*. Some signature samples of such category are displayed in Fig 13a. Note that they are not complex enough; moreover, as shown in [3,5,6], those signatures are highly variable. In the literature, such writers are actually considered as being “*problematic signers*” [25]. On the other hand, “*Sheep*” that are defined as writers easy to recognize, *correspond to writers of both medium and low Personal Entropy categories* (Fig 13b and 13c). These writers lead to a considerably lower FRR with the two classifiers as shown in Figs 9 and 10. Note that signatures of such writers are of higher complexity and also more stable as shown in [4,6].

**Fig 14:**
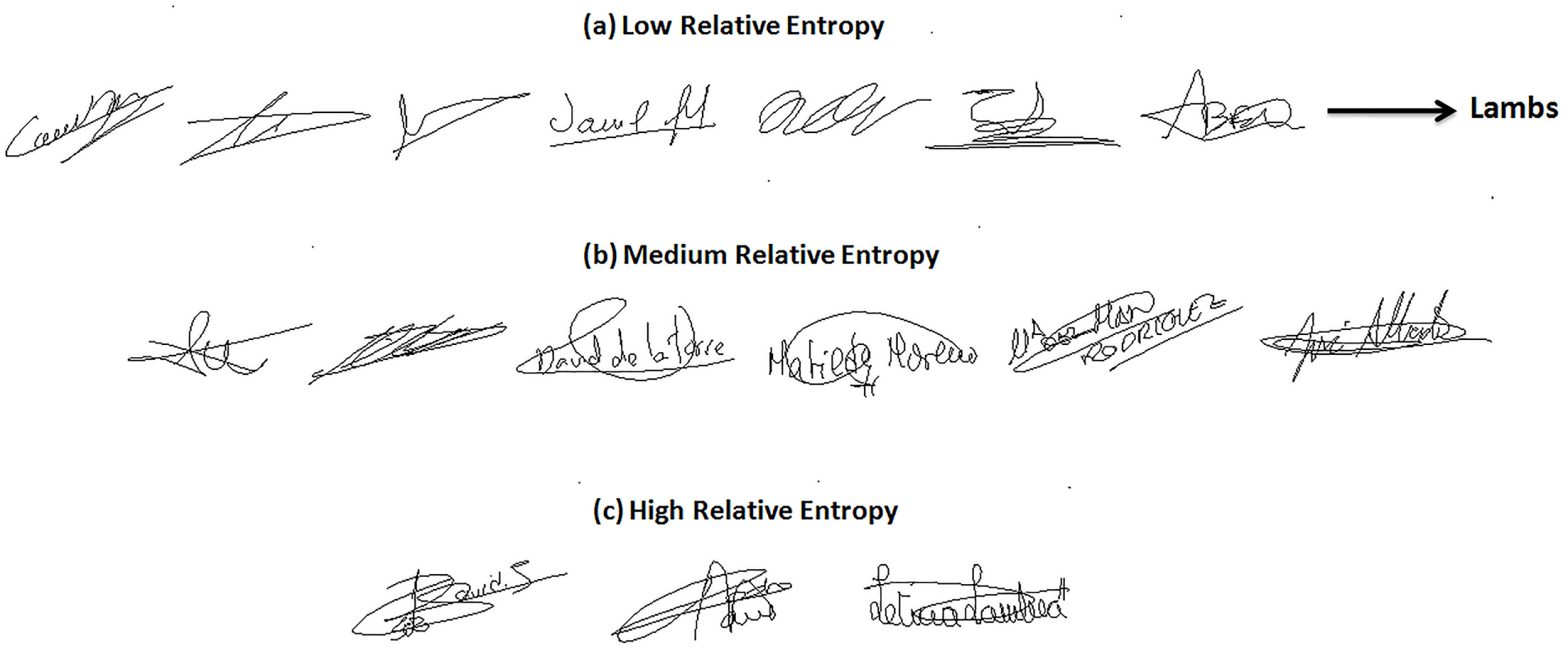
Signatures from MCYT-100 database of (a) Low, (b) Medium and (c) High Relative Entropy.

Finally, “*Lambs*” that are defined as writers that are easy to imitate, *correspond to writers of the lowest Relative Entropy category*. Indeed, for such writers, the Kullback-Leibler distance between genuine signatures and skilled forgeries is the lowest. Fig 14a displays signature samples of lowest Relative Entropy: such signatures are of lower complexity compared to the other categories shown in Fig 14b and 14c. Figs 11 and 12 confirm that such writers exhibit a higher FAR with the two classifiers, for most values of the decision threshold, especially when compared to the highest Relative Entropy category.

Note that *“Wolves”*, defined as writers successful at imitating (good forgers), do not appear in our analysis. In all previous works in the literature on the Biometric Menagerie, impostor scores are computed considering *only “random” forgeries* because most databases of physiological biometrics do not contain forgeries that could be qualified as being *“skilled”*. This is due to the difficulty of producing *skilled* attacks in the case of non-behavioral biometrics. This choice of “*random*” forgeries as the only type of attacks to biometric systems, allows supplying all the necessary information about the forger, since the latter is another client of the biometric system. But this choice on the type of attacks is totally *unrealistic* for the signature biometrics, which is actually challenged since a long time by skilled forgeries. For this reason, in this work, we chose to study the Signature Menagerie considering only these *high-level attacks*.

### 4.2. Hunting animals of Yager & Dunstone’s Menagerie

For analyzing the extended Menagerie of Yager & Dunstone [12], we propose to relate Personal Entropy and Relative Entropy by simply *overlapping* the two obtained automatic categorizations (one per entropy measure) on the same 100 writers of the MCYT-100 database. Indeed, we follow the same methodology used by Yager & Dunstone when defining new groups of animals in terms of a *relationship* between genuine and impostor scores [12].

**Fig 15:**
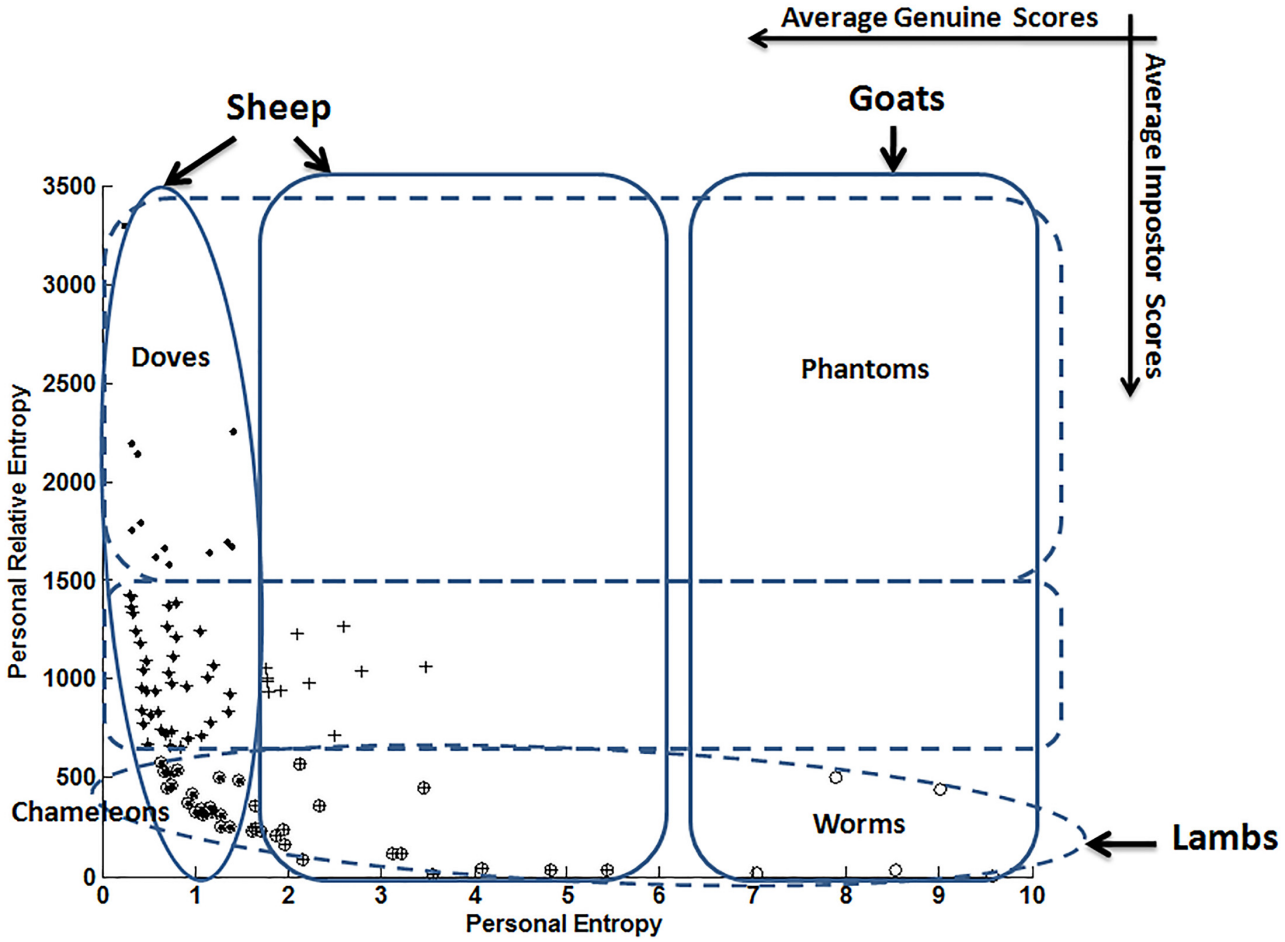
Yager & Dunstone’s Menagerie by overlapping Personal Entropy and Relative Entropy categories.

Fig 15 displays the resulting categories when *overlapping* the three Personal Entropy-based categories and the three Relative Entropy-based categories. We recall that categories are generated automatically by a clustering procedure and thus Fig 15 displays the *resulting membership* of users to different animal groups. For a better understanding, we also report at the top right corner in Fig 15 the axis of “Average Genuine Scores” and “Average Impostor Scores” that characterize the graphical representation of the Biometric Menagerie.

Additionally to *“Goats”*, *“Sheep”* and “*Lambs*” already analyzed in Section 4.1, three new sub-categories of Yager & Dunstone’s Menagerie emerge in Fig 15: “*Worms*”, “*Chameleons*” and “*Doves*”.

Let’s start by the two extreme sub-categories, namely *“Worms”* and *“Doves”*. On one hand, *“Worms”* appear as the worst writers as they are difficult to recognize (low genuine scores) and at the same time easy to forge (high impostor scores). Indeed, as shown in Fig 15, *“Worms”* are a sub-category of *“Goats”* and *“Lambs”*. In terms of entropy measures, “*Worms*” have the highest Personal Entropy and the lowest Relative Entropy. In MCYT-100 database, “*Worms*” represent only 5% of writers of the database.

On the other hand, “*Doves*” are the best writers since they are easy to recognize (high genuine scores) and at the same time difficult to forge (low impostor scores), as shown in Fig 15. In terms of entropy measures, “*Doves*” have the lowest Personal Entropy and the highest Relative Entropy. Actually, *“Doves”* are a sub-category of *“Sheep”*; they represent 12% of the MCYT-100 database.

Finally, “*Chameleons”* belonging to the intersection of *“Sheep”* and *“Lambs”* are easy to recognize (high genuine scores) and at the same time easy to forge (high impostor scores). In terms of entropy measures, “*Chameleons*” have the lowest Personal Entropy and the lowest Relative Entropy; they represent 17% of the MCYT-100 database.

The category of “*Phantoms*” that is a sub-category of *“Goats”* (low genuine scores and low impostor scores) is empty in Fig 15. In other biometrics (iris, face,…), “*Phantoms*” emerge when there is a failure at the acquisition of enrolment data (non-cooperative user, occlusions in images, dark or blurred images,…). In such cases, the poor quality of enrolment data generates at the matching step the rejection of both client and impostor data (random forgeries). For online signature, any degradation at the acquisition step appears as an intrinsic highly variable signature (case of *“Goats”*). In this case, contrary to other biometrics, although genuine samples tend to be rejected, a *“skilled”* forgery *may match the target signature better than a genuine one*.

We have this far obtained all the categories of the Biometric Menagerie with an alternative methodology exploiting quality measures for online signatures. In the following, we pursue our analysis by assessing performance *category per category* (*“Goats”*, *“Sheep”*, *“Lambs”*, *“Worms”*, *“Doves”*, *“Chameleons”*), and that with the local and global classifiers. The focus will be put on *relative performance assessment* between categories, and on the stability of results for both types of classifier, which are based on *different matching paradigms*.

**Fig 16:**
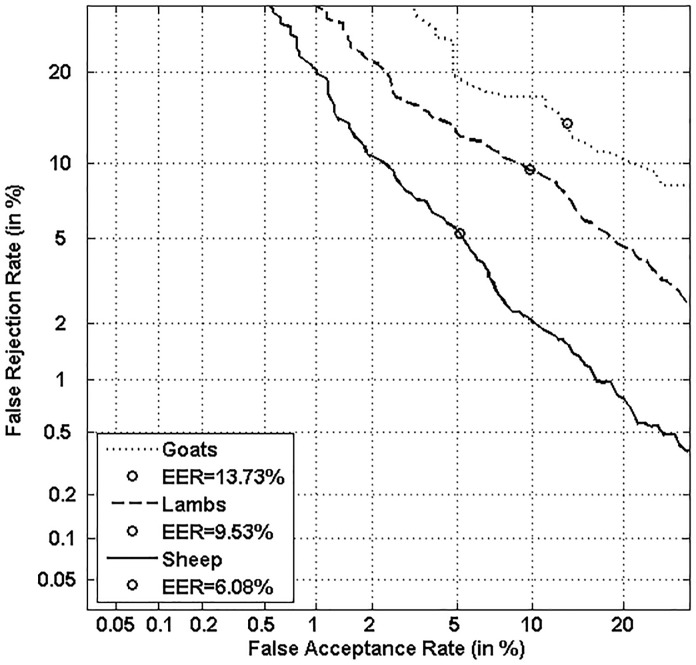
Performance on “Sheep”, “Goats” and “Lambs” with the local classifier.

**Fig 17:**
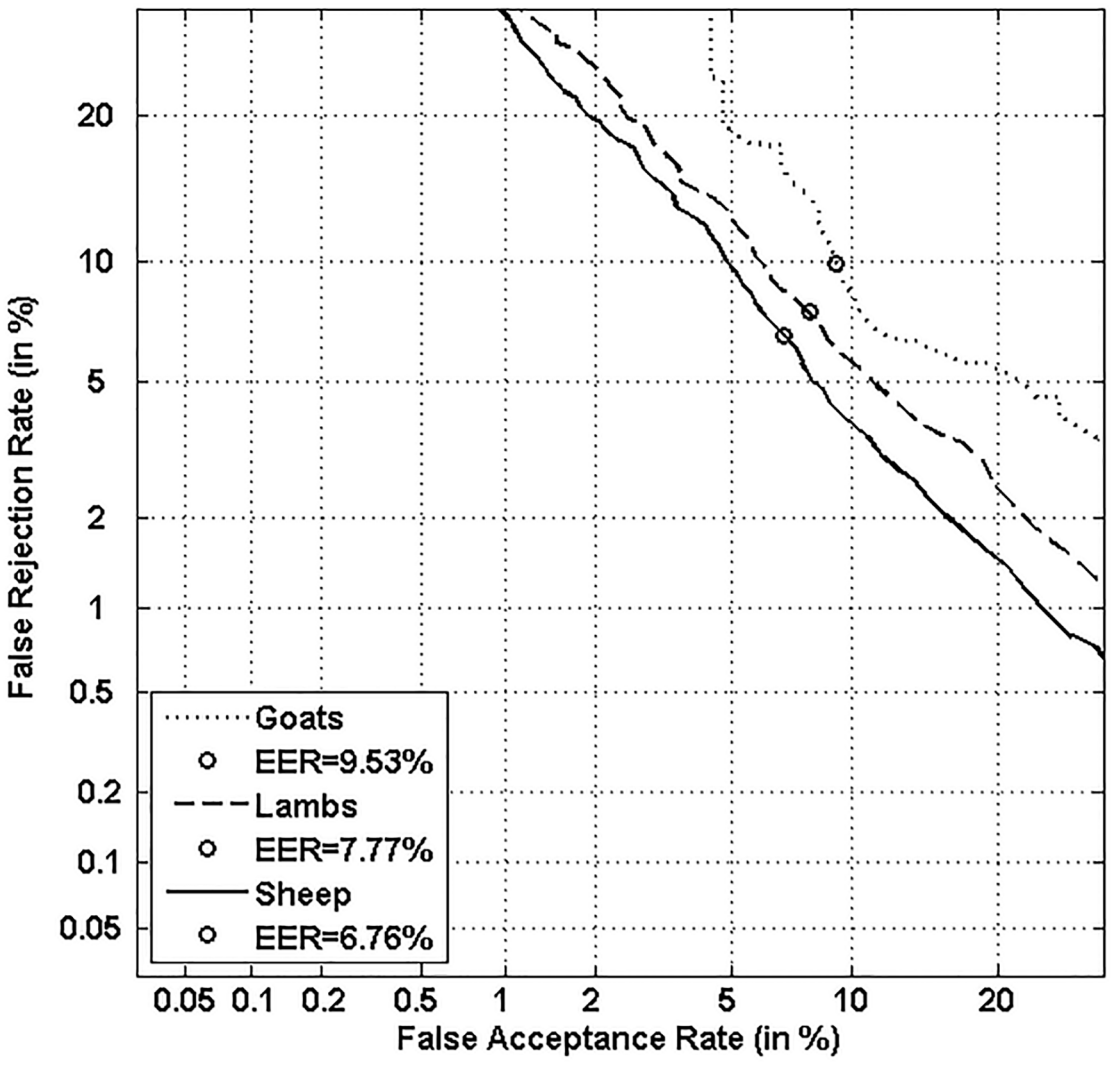
Performance on “Sheep”, “Goats” and “Lambs” with the global classifier.

**Fig 18:**
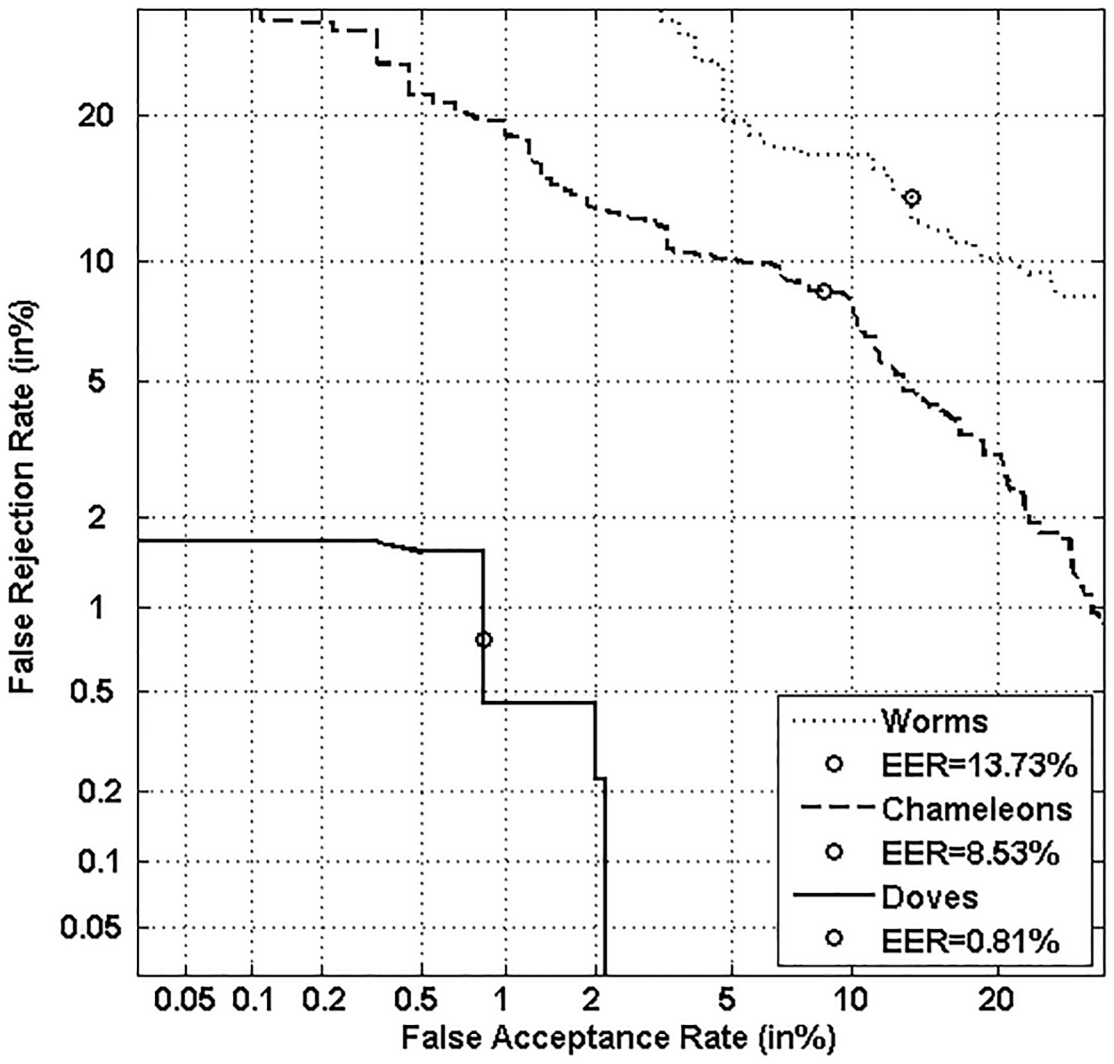
Performance on “Worms”, “Doves” and “Chameleons”, with the local classifier.

**Fig 19:**
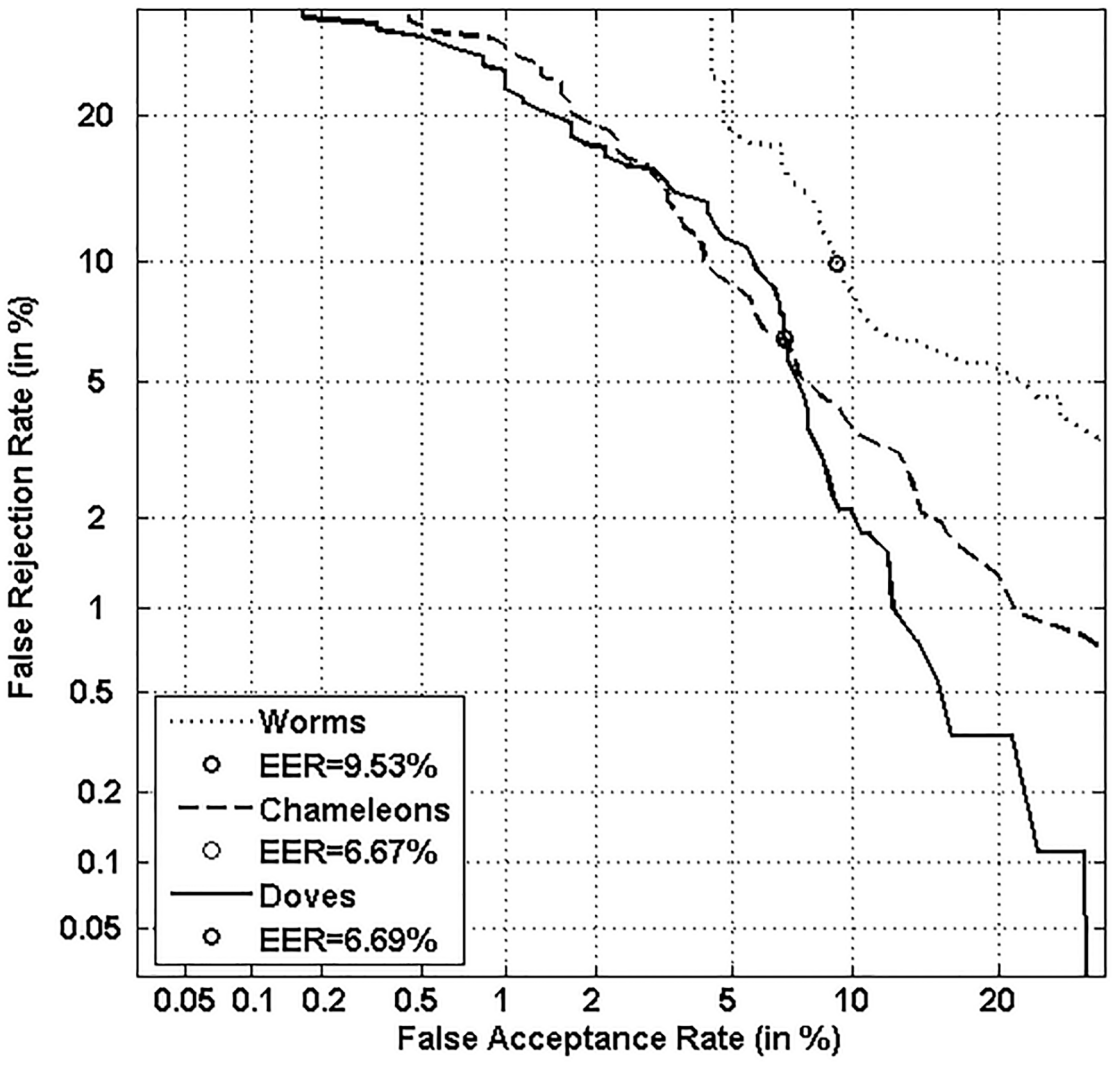
Performance on “Worms”, “Doves” and “Chameleons”, with the global classifier.

Figs 16 and 17 display classifier performance on the three categories of Doddington’s Menagerie, namely “*Goats*”, “*Sheep*” and “*Lambs*” with both classifiers. Figs 18 and 19 display classifier performance on the three sub-categories of Yager & Dunstone’s Menagerie, namely “*Worms*”, “*Doves*” and “*Chameleons*” with both classifiers.

We first observe that “*Goats*” and “*Sheep*” respectively give the worst and best performance for all functioning points and that for both classifiers. In case of the local classifier, a relative improvement of 55.71% at the EER is observed between the two categories. Such an improvement is of 29.06% for the global classifier. This result is natural since *“Sheep”* are writers that are easy to characterize while *“Goats”* are writers that are difficult to characterize [3,4,6].

Finally, “*Lambs”* show a performance level in between those of “*Sheep*” and “*Goats*”: indeed, as Doddington’s categories are non-exclusive (Fig 1), the category of “*Lambs”* contains both “*Sheep*” and “*Goats*”, but with a higher percentage of “*Sheep*” which improves performance for “*Lambs*” comparatively to *“Goats”*.

Concerning the retrieved sub-categories of Yager & Dunstone’s Menagerie (“*Doves*”, “*Worms*”, and “*Chameleons*”), Figs 18 and 19 show that the two extreme categories, “*Worms”* (the worst writers) and “*Doves”* (the best writers), *respectively give the worst and best performance for all functioning points and that for both classifiers*. In case of the local classifier, a relative improvement of 75% at the EER is observed between such two categories. Such an improvement is of 29.81% at the EER for the global classifier.

Moreover, when comparing Figs 18 and 19 to Figs 16 and 17, we notice as expected that performance of the two classifiers is degraded on “*Sheep*” relatively to that obtained on “*Doves*”. Finally, classifier performance on “*Chameleons”* is, as expected, in between those of “*Doves*” and “*Worms*”; indeed, “*Chameleons*” are easy to recognize as “*Doves*”, and at the same time easy to forge as *“Worms”*.

We have this far shown that our entropy measures allow retrieving *automatically* animal groups of the Biometric Menagerie by means of a clustering procedure. Indeed, we confirmed by means of two classifiers, that *the so obtained categories behave as expected in terms of relative verification performance*, *according to their description in the Biometric Menagerie* [11,12,13].

This study allows concluding that Relative Entropy combined to Personal Entropy may be used as an alternative for retrieving *automatically and directly from genuine and impostor signature samples*, writer categories of the Biometric Menagerie.

## 5. Conclusions and Perspectives

This work tackles for the first time the existence of animal groups of the Biometric Menagerie in the framework of online signature. Up to now, the concept of Biometric Menagerie was illustrated for other biometrics (speech, iris, fingerprint, face, …), and that relying on *classifiers’ average output scores*. This fact raises an inherent methodological difficulty pointed out by several authors [12,13,15,16,17,18,19]: *categories of the Menagerie are closely tied to both the matching algorithm and the dataset being used*.

The present work responds to this main issue by proposing an alternative methodology for hunting animals of the Biometric Menagerie. Our proposal is based on *quality measures* instead of on a classifier’s output scores. Indeed, quality measures have the main advantage of operating directly on *signature samples* and thus characterize writers independently of their behavior with respect to a specific classifier.

Our main contribution in this paper consists in tying two quality measures for signatures to the existing categories of the Biometric Menagerie: Personal Entropy, already presented in our previous works [2,3,5,6] and Relative Entropy, here introduced.

In this novel context for hunting animals of the Menagerie, we *coupled quality measures to an unsupervised clustering procedure*. This approach allows retrieving *automatically* animal groups and has the advantage of avoiding the use of thresholds for separating users into categories.

Through a progressive analysis, we first showed that the categories of Doddington’s Menagerie can be obtained by considering *separately* Personal Entropy and Relative Entropy. Indeed, “*Goats*” and “*Sheep*”, defined in Doddington’s Zoo in terms of FRR only [11], can be retrieved with Personal Entropy that operates only on *genuine* signature samples, while “*Lambs*” defined in terms of FAR only [11], can be retrieved with Relative Entropy that operates on *both genuine and impostor* signature samples.

Then, analogously to Yager & Dunstone’s methodology [12,13], by *combining the two quality measures*, we retrieved extra categories of the extended Zoo of Yager & Dunstone (*“Chameleons”*, *“Doves”*, *“Worms”*).

Our study showed on the widely used MCYT-100 online signature database, that the majority of writers behave as “*Sheep*” (95% of the database). This result is in perfect accordance with Doddington’s definition of *“Sheep”* [11]: *“Sheep dominate the population”*. On the other hand, “*Worms*”, defined by Yager & Dunstone as the worst conceivable users of a biometric system [13], represent only 5% of the database. These figures support the use of online signature as a reliable behavioral biometric trait *for most individuals*. This result is particularly interesting in the framework of Biometrics since contrary to other modalities, we considered a difficult type of forgeries, namely *skilled forgeries*.

For a better insight on the meaning of the obtained categories, we also carried out *an extensive performance analysis on animal groups*. To this end, two classifiers based on different matching paradigms (local versus global approaches) were exploited. Our experimental study reveals that the obtained categories *behave as expected in terms of relative verification performance*, *according to their description in the Biometric Menagerie* [11,12,13].

We have this far proved the *existence of the Biometric Menagerie for online signature*. Also, our original methodology based on measuring signature quality revealed that *animal groups exist beyond any classifier that could be used*. These new results impact online signature verification since on a given database, animal groups can now potentially be used to compare different signature verification approaches and find out which is the most appropriate for each animal group. Furthermore, our approach could be useful in the framework of signature competitions: on one hand, an analysis could be conducted on the development and test datasets about *how well user groups are represented*; on the other hand, performance should be assessed *per category*.

Moreover, the proposed Relative Entropy measure presents a great potential in many different directions:

1. It could be used to rank sets of forgery of different types (static, dynamic, synthetic) in terms of their proximity to the target genuine signatures for a more accurate performance assessment of signature verification systems, for instance in competitions.
2. As these last years, many interesting works on synthetic signature generation have been published, Relative Entropy measure could be exploited for assessing the quality of synthetic signatures. Indeed, it could be a quantitative indicator of how close a set of synthetic signatures are to a set of genuine signatures and that for a given writer.
3. Relative Entropy is based on the statistics of forgery production and thus presents a strong potential for analyzing who are “*Wolves*” in a population. Indeed, Personal Relative Entropy could be used for characterizing a given forger by estimating the local PdFs of the skilled forgeries that he/she produced.
4. The previous idea could be exploited at the acquisition step of online signature databases for selecting good forgers or even for training forgers to improve themselves.

In the future, we aim at extending our analysis on larger databases, for studying how the distribution of the population into animal groups evolves. This could indeed allow having a complete picture of who are the users of online signature verification systems.

## Supporting Information

S1 Appendix (DOCX)

S1 Fig The resulting dendrogram of the Hierarchical Clustering procedure on Relative Entropy values. (TIF)

S2 Fig Krzanowski-Laï index for each value of (*k*) number of writer categories. (TIF)

S3 Fig C-index for each value of (*k*) number of writer categories. (TIF)

S4 Fig RMSSTD Group indices for each value of (*k*) number of writer categories. (TIF)
