# Supplementary material for: On Hunting Animals of the Biometric Menagerie for Online Signature
Source: PLoS One. 2016 Apr 7;11(4):e0151691. doi: 10.1371/journal.pone.0151691 (PMC4824397; doi:10.1371/journal.pone.0151691)
Supplement: S1 Appendix — (DOCX) [file pone.0151691.s001.docx]

# Number of writer categories

The study on the optimal number of writer categories is carried out on Relative Entropy values, by means of the hierarchical clustering with “Ward’s linkage” [37,38]. This linkage function seeks to choose the successive clustering steps that minimize the increase of the distance between two clusters at each step. At each step of the Clustering (value of *k*, the number of clusters), different validity indices are computed: C-index [40], Krzanowski-Laï index [41], and 4 validity indices of the RMSSTD Group [39]. The procedure of identifying the optimal number of categories is as follows:

1. Apply the Hierarchical Algorithm on entropy values associated to each writer. Then, plot the “dendrogram” displaying the evolution of the objective function (a dissimilarity measure) according to the clustering carried out at each step.
2. By observing the important changes of the objective function in the dendrogram, infer the interval of variation of *k*, namely *k_min_* and *k_max_*
3. For each value of *k* between *k_min_* and *k_max_*, compute the validity indices.
4. For each validity index, plot the obtained value of the index as a function of *k*.
5. Based on this plot, the optimal number of clusters can be identified following two approaches [39]: if the validity index as a function of *k* is a monotonic function, the optimal number corresponds to the value of *k* at which a significant local change in value of the index occurs; otherwise, the optimal number is retrieved according to the intrinsic criterion of such an index:

- C-index should be minimized [40];
- Krzanowski-Laï index should be maximized [41];
- The validity indices of the RMSSTD Group should be used simultaneously; the optimal number of clusters corresponds to the values of *k* at which a significant variation of these indices is observed [39].

1. Finally, the number of optimal categories which is the most represented among all the considered indices is selected (majority voting procedure).

Following the above-mentioned methodology, we apply in the sequel, the cluster analysis on Relative Entropy values associated to writers of the MCYT-100 database. "S1 Fig" shows the resulting dendrogram.

**S1 Fig: The resulting dendrogram of the Hierarchical Clustering procedure on Relative Entropy values.**

The important changes of the objective function are represented by 2 dotted lines (2 “clustering levels”): at a value of 5.5 for the objective function corresponding to a partition of the data into 3 clusters and, at a value of 13 for 2 clusters. Therefore, we will compute the validity indices for *k* varying between 2 and 5 clusters.

**S2 Fig: Krzanowski-Laï index for each value of (*k*) number of writer categories.**

**S3 Fig: C-index for each value of (*k*) number of writer categories.**

**S4 Fig: RMSSTD Group indices for each value of (*k*) number of writer categories.**

"S2 Fig," "S3 Fig," and "S4 Fig." show the graphs of all the validity indices computed after clustering writers according to their Relative Entropy with the Hierarchical algorithm. For each index, the optimal number of clusters is indicated by a squared symbol on the validity index curve. We find that all validity indices indicate that the optimal number of clusters is *k*=3; indeed, in all graphs, the first knee corresponds to *k*=3. This study confirms that a choice of three Relative Entropy-based writer categories leads to the most homogeneous writer categories in terms of Relative Entropy, and thus in terms of signature complexity, variability and vulnerability to attacks by skilled forgeries.
