# Supplementary figures and images for: On Hunting Animals of the Biometric Menagerie for Online Signature
Source: PLoS One. 2016 Apr 7;11(4):e0151691. doi: 10.1371/journal.pone.0151691 (PMC4824397; doi:10.1371/journal.pone.0151691)

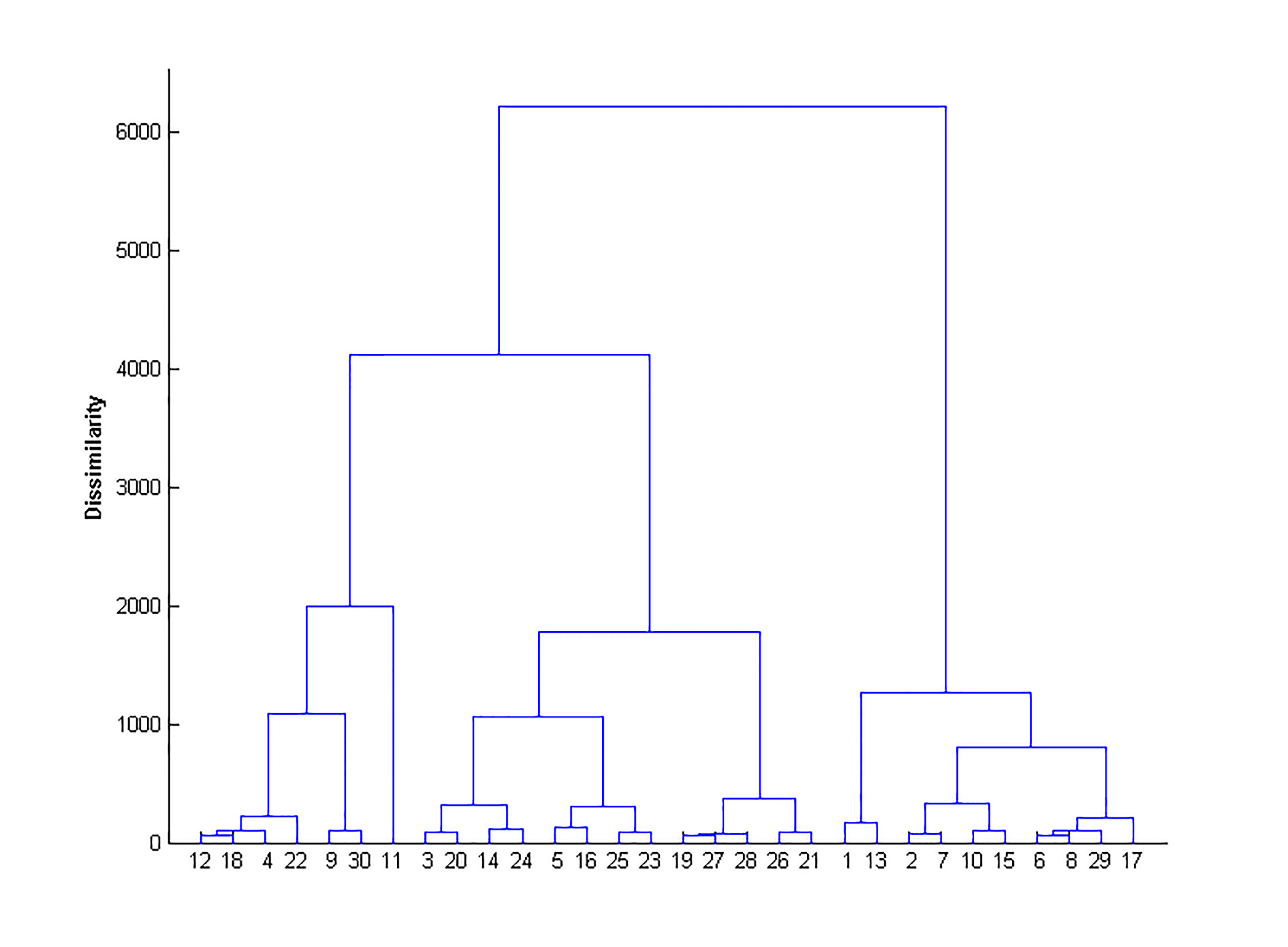

Supplement: S1 Fig — (TIF) [file pone.0151691.s002.tif]

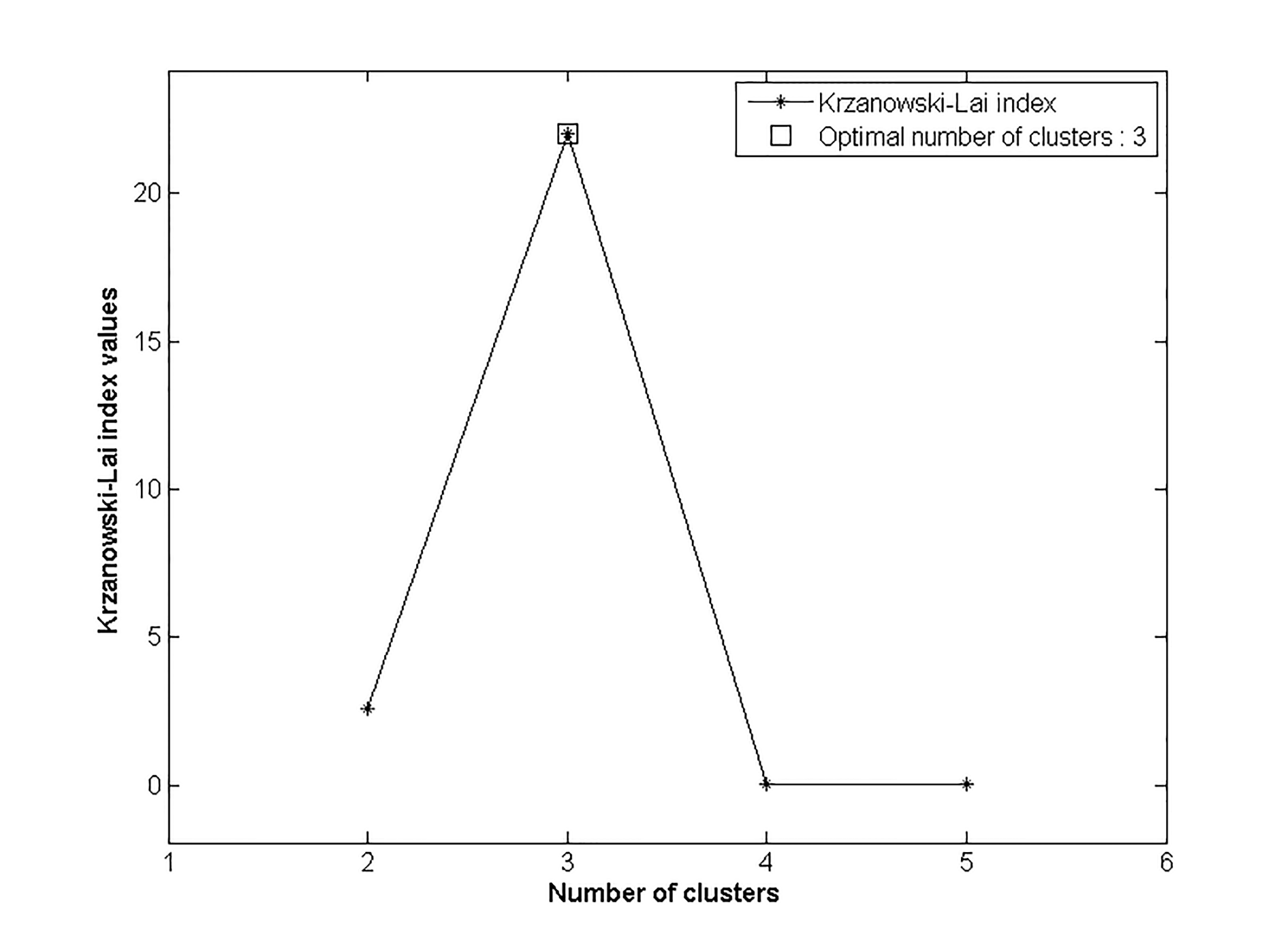

Supplement: S2 Fig — (TIF) [file pone.0151691.s003.tif]

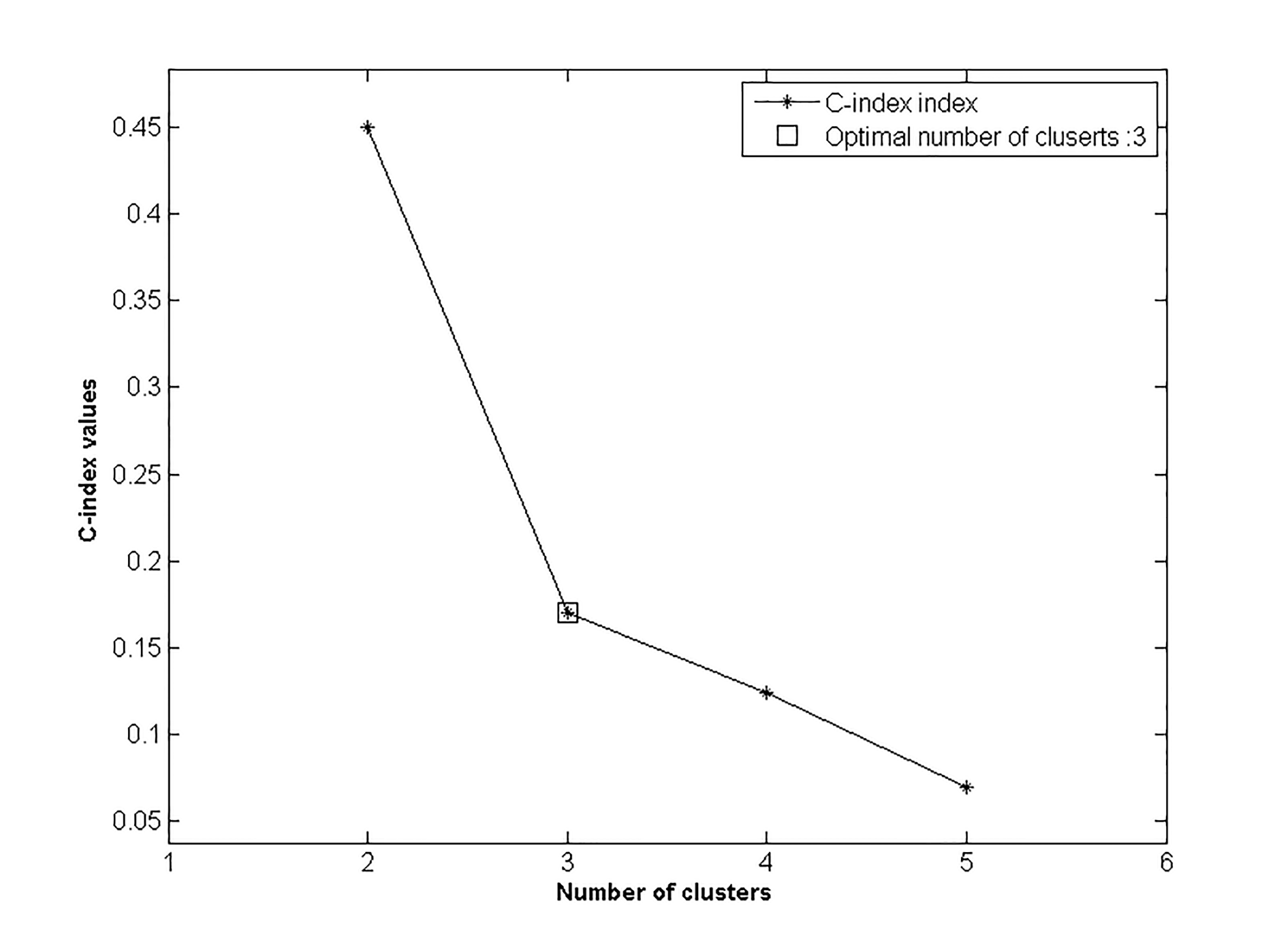

Supplement: S3 Fig — (TIF) [file pone.0151691.s004.tif]

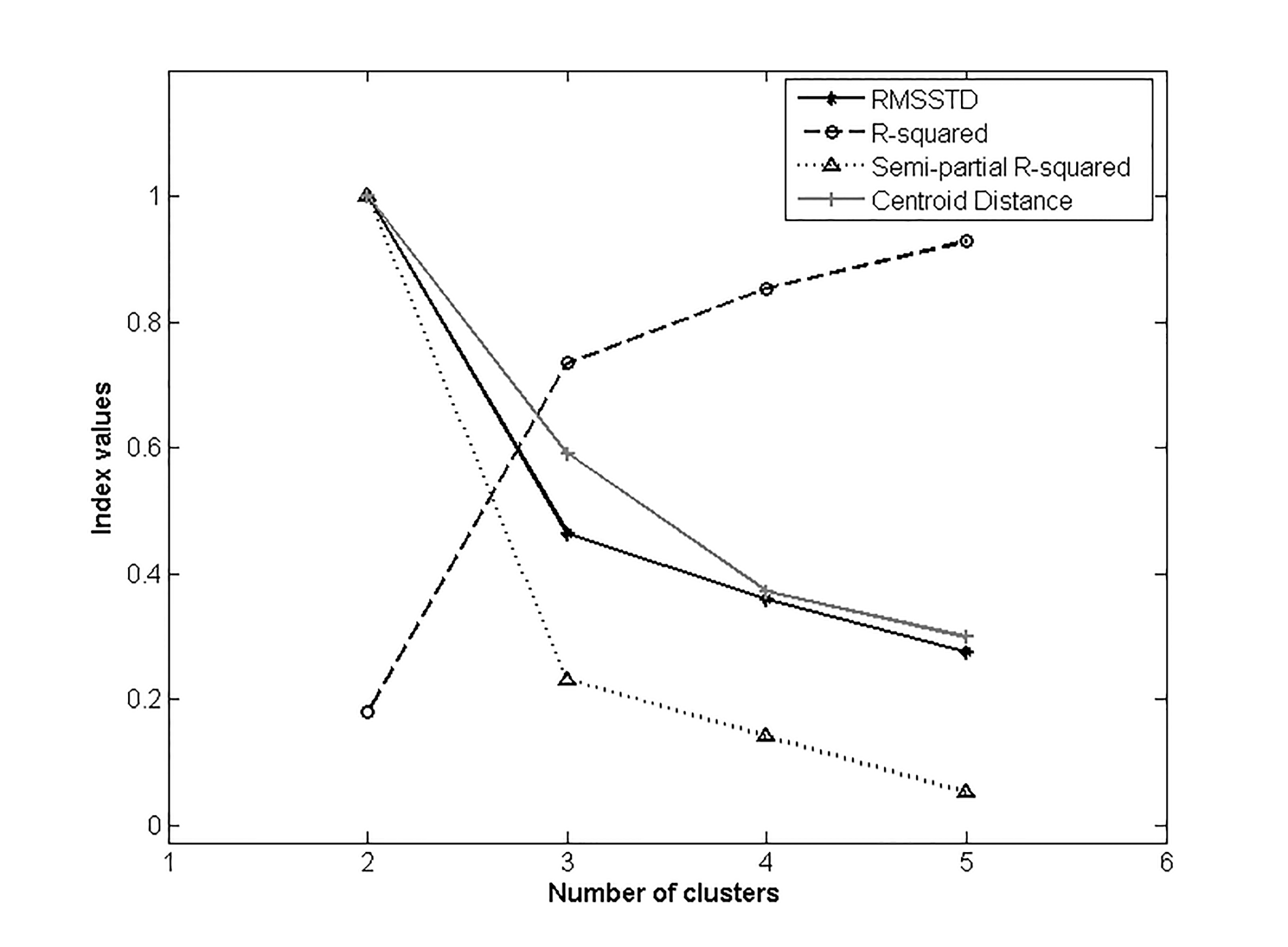

Supplement: S4 Fig — (TIF) [file pone.0151691.s005.tif]
